# Impact of Recording Conditions and Feature Fusion on Dementia Classification Using Spectral, Entropy, and Complexity EEG Features

**DOI:** 10.3390/e28091034

**Published:** 2026-09-20

**Authors:** Kerimay Sari, Samaneh Kouchaki, Daniel Abasolo

**Affiliations:** 1 Centre for Environmental and Biomedical Engineering, School of Engineering, University of Surrey, Guildford GU2 7XH, UK; d.abasolo@surrey.ac.uk; 2 Centre for Vision, Speech and Signal Processing (CVSSP), University of Surrey, Guildford GU2 7XH, UK; samaneh.kouchaki@surrey.ac.uk

**Keywords:** entropy, complexity, spectral features, recording conditions, EEG, Alzheimer’s disease, mild cognitive impairment

## Abstract

Resting-state electroencephalography (EEG) is a promising low-cost, non-invasive modality for supporting differential classification of Alzheimer’s disease (AD), mild cognitive impairment (MCI), and healthy controls (HCs). However, it remains unclear whether the combination of different EEG feature families consistently improves classification and whether their utility varies with recording condition. We compared spectral, entropy-based, and complexity-based features, both individually and in combination, for AD-HC, AD-MCI, and MCI-HC classification under eyes-closed (EC) and eyes-open (EO) conditions using an age-matched paired subset of the CAUEEG data set. Classification was evaluated using nested leave-one-subject-out cross-validation with two-stage hierarchical LightGBM gain-based feature selection. Under the EC condition, the highest balanced accuracies (BAs) were obtained with spectral features for AD-HC (79.99%), spectral–entropy for AD-MCI (68.33%), and spectral–entropy–complexity for MCI-HC (63.15%). Under the EO condition, spectral features performed best for AD-HC (84.91%) and AD-MCI (66.90%), whereas entropy performed best for MCI-HC (70.14%). Feature fusion therefore showed task-dependent rather than consistent benefits. Although numerical EC-EO differences were observed, none remained significant after paired permutation testing. Overall, the results suggest that the relative usefulness of EEG feature families varies across diagnostic comparisons and recording conditions.

## 1. Introduction

According to estimates based on the Global Burden of Disease Study, the number of people living with dementia worldwide is expected to rise from 57.4 million in 2019 to 152.8 million in 2050 [1]. It is also known that Alzheimer’s disease (AD) accounts for approximately 70% of dementia cases [2]. In this context, AD is the most common form of dementia, especially among people aged 65 years and older [1].

AD is a neurodegenerative disorder that progressively and irreversibly impairs cognitive function, memory, behaviour, and activities of daily living [3]. Although treatments that may slow AD progression are available, there is currently no cure [4]. Furthermore, initiating AD treatment at a late stage of the disease, when significant brain damage has already occurred, may limit treatment efficacy [4]. Therefore, early diagnosis is of paramount importance, as it may enable timely interventions that help slow disease progression whilst providing patients and their families with valuable time for advance care planning and disease management.

Mild cognitive impairment (MCI) is a prodromal stage between normal ageing and AD, in which cognitive impairments begin but symptoms are not as severe as in AD [5]. Approximately 10–15% of MCI patients convert to AD each year [6]; furthermore, long-term follow-up studies have reported that this conversion rate increases over time, with approximately 33% of patients converting to AD within 5 years [7]. One of the major challenges in diagnosing AD and MCI is normal ageing [8]. Since cognitive changes seen in old age may resemble disease symptoms, symptoms may sometimes be considered normal ageing, leading to delayed diagnosis.

Neuropsychological clinical tests such as the Mini-Mental State Examination (MMSE); Alzheimer’s Disease Assessment Scale—Cognitive Subscale (ADAS-Cog) [3,9]; and Clinical Dementia Rating (CDR) [10] are commonly used in the diagnosis of AD. Magnetic resonance imaging (MRI) and positron emission tomography (PET) are used as neuroimaging methods [11]. In addition, cerebrospinal fluid (CSF) biomarker analysis, which provides pathological information in AD, is one method used to examine amyloid- and tau-related pathophysiological changes in the brain [12]. However, neuropsychological tests often lack specificity for AD, as they are primarily designed to evaluate global cognitive decline and dementia severity. Although neuroimaging methods such as MRI and PET provide valuable diagnostic information, they are not readily applicable to all patients, due to high cost and limited accessibility. CSF analysis, on the other hand, has significant limitations for routine clinical application and repeated measurements, particularly in older people, due to its invasive nature.

As an alternative to these methods, electroencephalography (EEG) is a non-invasive, low-cost, readily accessible, and highly portable measurement method that can be applied under normal resting-state conditions. EEG records the brain’s electrical activity with high temporal resolution in the millisecond range, enabling non-invasive assessment of dynamic and functional changes in neuronal activity associated with AD and MCI.

A major EEG spectral hallmark of Alzheimer’s disease (AD) is EEG slowing, reflected by a redistribution of power from higher-frequency bands, including alpha, beta, and gamma, towards lower-frequency delta and theta bands. Although comparable slowing may occur during normal ageing, this pattern is typically more pronounced in AD and is associated with disease severity [2,9,13]. Furthermore, previous studies have shown that increases in the theta/alpha ratio [14] and the theta/gamma ratio [15] are associated with disease severity and cognitive impairment.

In addition to spectral alterations, several studies have shown that reduced inter-neuronal interactions, associated with neuronal loss in AD, may lead to EEG signals that are more predictable, more regular, and less complex [16,17,18]. Entropy and complexity algorithms can be used to quantify these dynamic changes. Entropy measures generally reflect signal irregularity and unpredictability, whereas complexity measures provide complementary information on temporal organisation, structural richness, and dynamic patterns in neural activity.

Despite the growing use of EEG to differentiate among AD, MCI, and healthy control (HC) groups, a key methodological gap remains in the selection and combination of EEG feature sets for classification. Many studies either focus on a single feature family (e.g., spectral, entropy-based, or complexity-based features) or combine multiple feature types without systematically evaluating whether and under what conditions such fusion improves classification performance.

Spectral, entropy-based, and complexity-based features capture distinct neurophysiological dimensions of EEG signal and may therefore provide complementary information when combined. However, their relative contributions may vary across diagnostic comparisons (e.g., AD-HC, AD-MCI, and MCI-HC) and recording conditions. Because eyes-closed (EC) and eyes-open (EO) states modulate resting-state EEG signal dynamics differently, the discriminative utility of each feature family may vary across recording protocols.

In this study, we evaluated spectral, entropy-based, and complexity-based EEG features, both individually and in combination, for AD-HC, AD-MCI, and MCI-HC classification under EC and EO conditions. We hypothesised that feature fusion would improve classification performance by integrating complementary EEG signal information across different feature families, and that these improvements would be condition-dependent, varying across both resting-state conditions and diagnostic comparisons. Accordingly, this study aimed to determine whether fused feature sets provide advantages over individual feature groups and to identify the feature families, brain regions, and electrodes most relevant to each classification task. To achieve this, we systematically compared spectral, entropy-based, and complexity-based EEG feature families under both eyes-closed (EC) and eyes-open (EO) resting-state conditions; evaluated whether feature fusion provides consistent or condition-specific benefits across AD-HC, AD-MCI, and MCI-HC classification tasks; and examined the regional and electrode-level distribution of the selected features to better understand how discriminative EEG information varies across diagnostic groups and recording conditions.

## 2. Materials and Methods

### 2.1. Data Set

The CAUEEG (Chung-Ang University Hospital EEG) data set [19] is a publicly available data set comprising 1379 recordings from 1155 patients, collected between 24 August 2012 and 12 March 2020. Recordings were acquired using a 21-channel configuration based on the International 10–20 system. Nineteen of these channels consist of standard EEG electrodes (Fp1, F3, C3, P3, O1, Fp2, F4, C4, P4, O2, F7, T3, T5, F8, T4, T6, Fz, Cz, and Pz), while the remaining two channels contain ECG and photic stimulation signals. The data were bandpass-filtered between 0.5 and 70 Hz and digitised at a sampling rate of 200 Hz using a digital electroencephalograph system (Comet AS40 amplifier EEG GRASS; Telefactor, Conshohocken, PA, USA).

Although the CAUEEG data set includes recordings from patients with a wide range of neurological conditions, the present study focused on three diagnostically relevant groups: AD, MCI, and HC. To minimise potential confounding by age, the subsequent analyses were performed on an age-matched subset drawn from these groups.

### 2.2. Preprocessing

All preprocessing steps were performed in MATLAB (R2025b) using the EEGLAB toolbox (2025.0.0) and the ASR, ICA (runica), and MARA plugins. The ASR-based clean_artifacts procedure was used to attenuate transient, high-amplitude bursts while preserving the underlying neural signal structure. Bad channels identified by ASR were reconstructed using spherical interpolation based on their spatial locations. This approach ensured a consistent number of channels and spatial configuration across all participants, thereby maintaining dimensional consistency for subsequent analyses.

Following ASR, a bandpass finite impulse response filter of order 660 with cut-off frequencies at 1 and 50 Hz was applied to suppress low-frequency drift and high-frequency noise, including muscle activity and 60 Hz line interference. The data were then decomposed using ICA (runica), and the MARA plugin was used to automatically classify independent components by their likelihood of representing artefacts. Components with an artefact probability > 0.80 were discarded to objectively remove non-neural components.

To ensure data integrity, a two-stage exclusion strategy was applied: recordings were excluded if more than three channels required ASR-based interpolation or if more than half of the independent components were classified as artefacts by MARA. This rigorous screening minimised the inclusion of irreparably corrupted data, thereby reducing noise and limiting the introduction of non-neural biases into the feature space.

### 2.3. Segmentation

Because the CAUEEG data set recordings included various conditions, such as EO, EC, and photic stimulation, EO and EC segments were extracted using event markers and then segmented into 5 s epochs. This epoch length was chosen to ensure adequate frequency resolution (0.2 Hz) for spectral analysis while providing sufficient sample points (1000 samples) for reliable estimation of entropy-based measures.

To enhance inter-subject comparability, the data set was standardised by selecting 20 epochs (100 s of EEG data) per participant. This threshold ensured a uniform amount of artefact-free data across subjects. It also provided sufficient data for robust estimation of spectral and entropy-based features while preserving the study sample size. Rather than employing stochastic selection or simple amplitude thresholding, epochs were selected using a spectral similarity approach to capture each subject’s most stable EEG profile, as described in [20]. For each channel, an epoch-by-epoch similarity matrix was constructed using Spearman’s rank correlation of power spectral density (PSD) vectors. A ‘global epoch score’ was derived by averaging these similarity indices across all channels, and the top 20 epochs with the highest global epoch scores were retained for analysis. This procedure filters out non-stationary artefacts and outliers that deviate from the subject’s typical spectral profile, improving the reliability, reproducibility, and signal-to-noise ratio of the extracted spectral metrics.

We created age-matched subsamples to minimise age-related confounding, and we observed no significant age differences between groups (Table 1). Subjects were randomly selected to ensure balanced age distributions across groups, minimising potential confounding factors. For EO/EC comparisons, a paired subset was defined so that EO and EC data represented two resting-state conditions from the same individuals. Table 1 presents the demographic characteristics and age-comparison statistics for the final paired EO/EC data set.

The 20 extracted representative epochs served as the basis for computing spectral, complexity, and entropy features, ensuring consistent, high-quality data input for each subject’s feature vector.

### 2.4. Feature Extraction

For each participant, feature extraction was performed on the 20 representative 5 s resting-state EEG epochs, with EO and EC conditions analysed separately.

Table 2 summarises the extracted features used in the classification analyses. We compared three main feature sets: spectral, entropy-based, and complexity-based features. In addition to evaluating each set separately, we also examined combined feature sets to assess whether they improved classification performance across diagnostic comparisons of multimodal EEG descriptors.

The selected feature sets were chosen to provide complementary descriptions of resting-state EEG activity from spectral, entropy, and complexity perspectives. Spectral features were included because they represent well-established descriptors of oscillatory brain activity and remain among the most widely used EEG features. In addition to conventional band-power measures, we extracted alpha-peak parameters, MF, and power ratios to capture distinct spectral properties of frequency-domain activity.

Entropy features were selected because entropy algorithms provide a powerful framework for quantifying the irregularity, uncertainty, and information content of EEG signals beyond what conventional spectral analysis can capture. Because no single entropy measure can comprehensively characterise all aspects of resting-state EEG dynamics, we included algorithms representing different theoretical formulations of entropy. Specifically, the entropy feature set comprised pattern-matching methods (FuzzyEn, RangeEn, and MSE) to quantify signal regularity; symbolic and ordinal methods (BubbleEn and WE) to characterise symbolic dynamics; information-theoretic measures (AE and EoE) to describe information content; and cross-signal similarity measures (XFuzzyEn) to assess non-linear inter-regional dependencies. Among these measures, AE and EoE are parameter-free entropy algorithms that, to the best of our knowledge, have not previously been evaluated for EEG-based AD classification. Together, these complementary entropy formulations enable a comprehensive evaluation of both established entropy measures and parameter-free entropy algorithms within a unified machine-learning framework for EEG-based AD classification.

Complexity features were selected to characterise the geometric, fractal, temporal, and dynamical properties of EEG signals, thereby complementing spectral and entropy-based descriptors. These measures provide additional information regarding signal organisation and temporal structure.

Feature computation was performed channel by channel, using 19 standard EEG electrodes. For each subject and channel, the 20 feature values across the 20 epochs were averaged, yielding a single representative mean value for each channel of each subject.

XFuzzyEn and mvMSE were exceptions to this channel-wise approach. XFuzzyEn was used to assess non-linear similarity between scalp regions. The 19 electrodes were grouped into five anatomical regions: frontal (Fp1, Fp2, F3, and F4), central (C3, Cz, C4, Fz, and Pz), left temporal (F7, T3, and T5), right temporal (F8, T4, and T6), and parieto-occipital (P3, P4, O1, and O2), yielding 10 inter-regional comparisons. For each regional pair, XFuzzyEn was calculated across all possible electrode-pair combinations formed by the electrodes within the corresponding regions and then averaged to obtain a single regional value per epoch, which was subsequently averaged across the 20 epochs for each subject.

For mvMSE, all 19 EEG channels were analysed jointly as a single multivariate representation to quantify temporal complexity across multiple time scales. mvMSE was calculated at nine scales (scales 2–10), yielding nine features per epoch. The values at each scale were subsequently averaged across the 20 epochs for each subject.

The following frequency bands were used for PSD-based spectral features: delta (1–4 Hz), theta (4–8 Hz), alpha (8–13 Hz), beta (13–30 Hz), and gamma (30–50 Hz), and total band (1–50 Hz).

Algorithm-specific parameter settings are summarised in the Supplementary Materials (Supplementary Table S1). Parameters were primarily selected based on previous publications, with selected modifications based on signal length and methodological considerations. No parameters were empirically optimised using the present data set; the literature basis and rationale for any modifications are provided in Table S1.

### 2.5. Feature Selection and Classification

**Figure 1:**
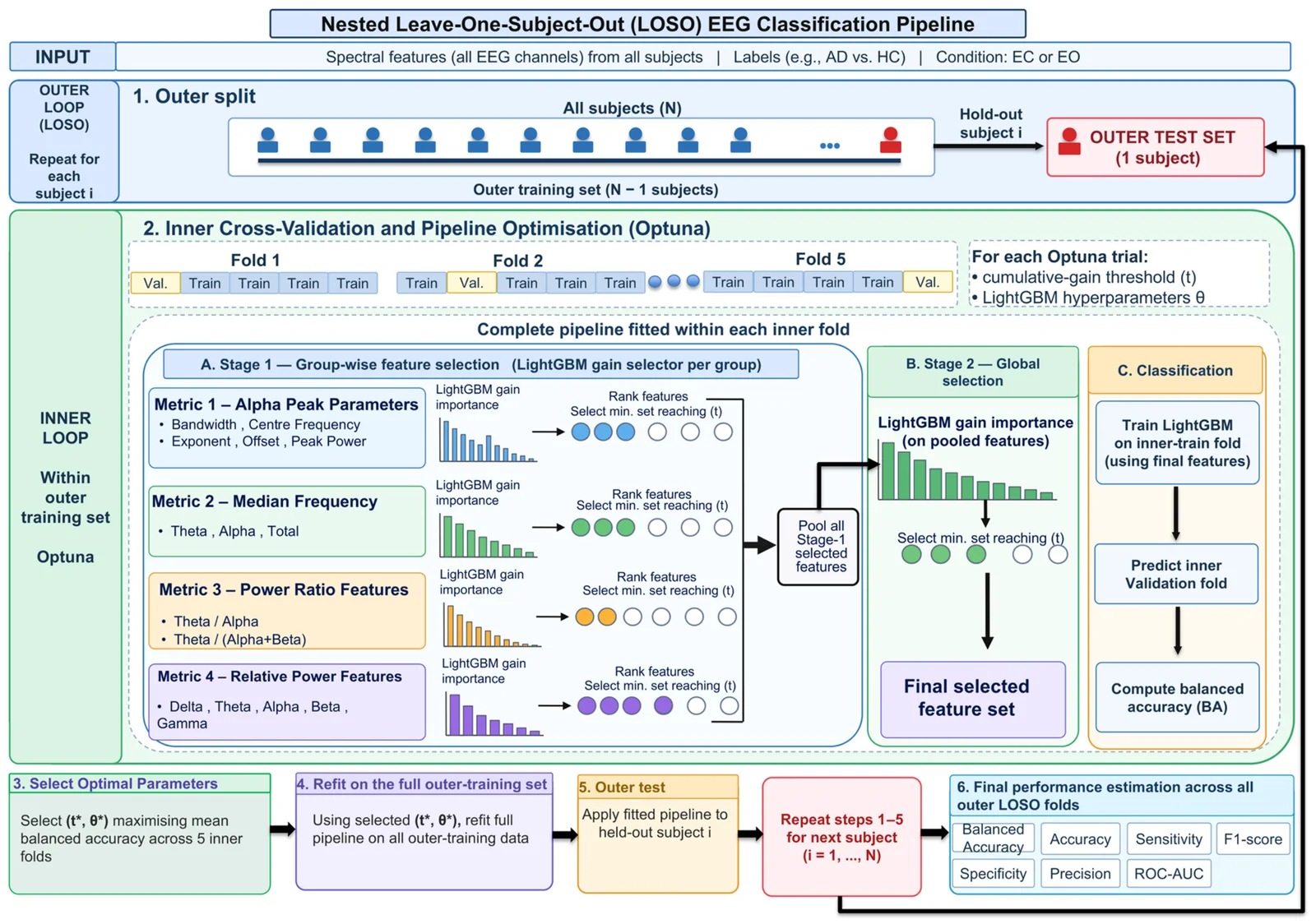
Nested LOSO framework for two-stage hierarchical LightGBM feature selection and classification, illustrated using spectral features. In each outer fold, feature selection and Optuna-based tuning are performed only on the training subjects, while the held-out subject is reserved for final testing. Stage 1 selects features within metric groups, and Stage 2 performs global selection on the combined features using the same cumulative-gain threshold (*t*). The optimised pipeline is retrained on the full outer-training set and evaluated on the held-out subject. Here, *t* denotes the cumulative-gain threshold, θ denotes the LightGBM hyperparameter set, and * indicates the selected candidate value.

As illustrated in Figure 1, a nested leave-one-subject-out (LOSO) cross-validation approach was employed to ensure a reliable and unbiased estimate of classification performance. To prevent information leakage, all feature selection and model-tuning steps based on data were conducted solely on the outer training set. In each outer LOSO iteration, one subject was held out as the outer test set, while the remaining *N* − 1 subjects formed the outer training set. Within the outer training set, stratified inner cross-validation with five folds was used to optimise the analysis pipeline. Consequently, the outer test subject was not involved in feature ranking, threshold setting, or hyperparameter tuning, and was only used for the final prediction in that fold.

The analysis used 40 predefined EEG metrics, including 8 complexity, 17 entropy, and 15 spectral metrics. Seven feature configurations were tested: complexity; entropy; spectral; all pairwise combinations (complexity and spectral; entropy and spectral; and complexity and entropy); and the combination of complexity, entropy, and spectral. These analyses were carried out separately for the EC and EO conditions and for the binary classification tasks of AD vs. HC, AD vs. MCI, and MCI vs. HC.

We used a two-stage hierarchical feature-selection framework based on LightGBM gain importance [40]. For feature ranking, LightGBM models were trained using fixed hyperparameters, a binary objective, and balanced class weights. The hyperparameters were set to n_estimators = 200, learning_rate = 0.05, max_depth = 3, num_leaves = 7, min_child_samples = 5, min_split_gain = 0.0, reg_alpha = 0.01, reg_lambda = 0.01, and colsample_bytree = 1.0. These models were used only for feature selection and were not hyperparameter-optimised. Features were ranked by gain importance in descending order. The gain values were then normalised by the total gain, and the smallest set of top-ranked features whose cumulative normalised gain reached the candidate threshold (*t*) was retained.

As shown in Figure 1, Stage 1 grouped related metrics separately instead of combining all features initially. Within the spectral family, 15 metrics were organised into four groups: alpha-peak descriptors (BW, CF, exponent, offset, and PW); three MF descriptors (theta, alpha, and total); two power-ratio descriptors; and five RP descriptors (delta, theta, alpha, beta, and gamma). For entropy features, MSE scales 2–10 were grouped together in Stage 1, while the remaining entropy metrics were treated as individual groups. This group-wise strategy was designed to minimise the disproportionate influence of feature groups of varying sizes and to prevent larger groups from dominating the subsequent global selection stage.

The eight complexity metrics were also treated as separate Stage-1 groups. In combined feature configurations, these family-specific grouping rules were preserved. The features retained in Stage 1 were then pooled and re-evaluated using a second LightGBM gain selector in Stage 2, so only features that retained both within-group selection and global selection entered the final classifier.

The cumulative-gain threshold was common to all the Stage-1 and Stage-2 selectors in each candidate pipeline. This threshold was optimised during inner cross-validation using candidate values of 0.80, 0.85, and 0.90. In the same inner-validation procedure, we simultaneously optimised the final LightGBM classifier’s hyperparameters using Optuna with a Tree-structured Parzen Estimator (TPESampler). For each outer fold, we ran 40 Optuna trials, including 10 start-up trials, and selected the configuration with the highest mean inner-fold balanced accuracy (BA). The search space for the classifier included n_estimators in {100, 200, 300}, learning_rate in {0.01, 0.05, 0.10}, max_depth in {2, 3, 4}, num_leaves in {3, 5, 7}, min_child_samples in {5, 10, 15}, reg_alpha in {0.0, 0.1, 0.5}, and reg_lambda in {0.0, 0.1, 0.5}. Only combinations satisfying num_leaves ≤ 2^max_depth were evaluated. The classifier used a binary objective and balanced class weights, with colsample_bytree set to 1.0. Early stopping was not used; instead, the number of boosting trees was optimised via n_estimators.

After identifying the optimal threshold and classifier hyperparameters, we refitted the complete two-stage feature-selection and classification pipeline on the full outer-training set. We applied it once to the held-out subject, repeated this procedure until every subject had served once as the outer test subject, and calculated the final performance only from the outer-loop predictions.

Classification performance was evaluated using BA, overall accuracy (ACC), sensitivity (TPR), specificity (TNR), precision, the F1-score, and receiver operating characteristic area under the curve (AUC). We used a probability threshold of 0.50 to assign the predicted class, and we estimated 95% confidence intervals for the performance measures using 1000 stratified bootstrap resamples. We selected BA as the main accuracy measure because it accounts for class imbalance by giving equal weight to the classification performance of both classes.

Performance metrics were derived from the confusion matrix, where true positives (TPs) denote positive samples correctly classified as positive, true negatives (TNs) denote negative samples correctly classified as negative, false positives (FPs) denote negative samples incorrectly classified as positive, and false negatives (FNs) denote positive samples incorrectly classified as negative. The true-positive rate (TPR) quantifies the proportion of actual positive samples that are correctly identified by the classifier and is calculated according to Equation (1).

(1) $$\begin{array}{c} \mathrm{TPR}=\frac{TP}{TP+FN} \end{array}$$

Similarly, the true-negative rate (TNR) measures the proportion of actual negative samples that are correctly identified by the classifier and is calculated according to Equation (2).

(2) $$\begin{array}{c} \mathrm{TNR}=\frac{TN}{TN+FP} \end{array}$$

BA is computed as the arithmetic mean of TPR and TNR, as given in Equation (3).

(3) $$\begin{array}{c} \mathrm{BA}=\frac{TPR+TNR}{2} \end{array}$$

ACC represents the proportion of correctly classified samples among all samples and is calculated according to Equation (4).

(4) $$\begin{array}{c} \mathrm{ACC}=\frac{TP+TN}{TP+TN+FP+FN} \end{array}$$

Precision, also referred to as positive predictive value, quantifies the proportion of samples classified as positive that are actually positive and is calculated according to Equation (5).

(5) $$Precision=\frac{TP}{TP+FP}$$

The F1-score provides a balanced measure of precision and sensitivity by calculating their harmonic mean. It is particularly useful when both false-positive and false-negative classifications need to be considered simultaneously and is calculated according to Equation (6).

(6) $$F1-score=\frac{2*(Precision*TPR)}{Precision+TPR}$$

The AUC was calculated using the predicted probabilities generated by the classifier. TPR and FPR were determined at various classification thresholds and plotted to construct the ROC curve. The area under this curve represents the AUC.

To assess the stability of feature selection across outer LOSO folds, the frequency with which features from each EEG electrode appeared in the final selected feature set was examined. For each diagnostic classification, recording condition, and feature set, we ran 5000 label permutations to assess whether the observed performance exceeded chance level. We shuffled the class labels randomly to build a null distribution of BA scores and then compared our observed score against that distribution to obtain an empirical permutation *p*-value. To address multiple comparisons, permutation *p*-values were adjusted using the Benjamini–Hochberg false-discovery rate (FDR) method. FDR-adjusted *p*-values (*q*-values) below 0.05 were considered statistically significant.

## 3. Results

### 3.1. Overall Classification Performance

Classification results are presented separately for the EC and EO conditions across the three binary diagnostic comparisons and seven feature configurations. The results are subsequently compared across feature families and recording conditions, followed by an analysis of feature-selection stability.

### 3.2. Eyes-Closed Classification Performance

**Figure 2:**
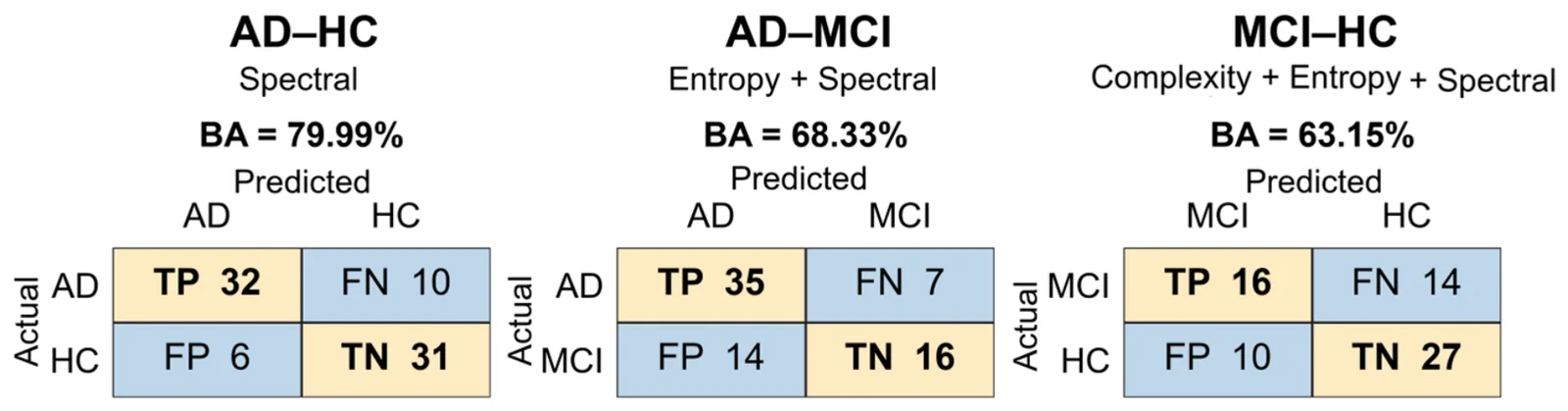
Confusion matrices for the highest BA observed in each diagnostic comparison under the EC condition. Rows indicate the actual class, and columns the predicted class.

Under the EC condition, classification performance varied considerably across diagnostic comparisons. The AD-HC comparison demonstrated the highest level of discrimination, followed by AD-MCI and MCI-HC. The feature configuration yielding the highest BA also differed by task: spectral features alone yielded the highest BA for AD-HC, the spectral–entropy combination for AD-MCI, and the combined spectral–entropy–complexity feature set for MCI-HC. Notably, spectral features were represented in the highest-performing configuration for all three comparisons. Figure 2 presents the confusion matrices corresponding to the feature configuration that yielded the highest BA in each diagnostic comparison.

Based on these confusion matrices, Table 3 summarises the corresponding performance metrics for the best-performing feature configuration in each diagnostic comparison. Results for all seven feature configurations are provided in the Supplementary Materials (Supplementary Table S2).

#### 3.2.1. AD vs. HC

**Figure 3:**
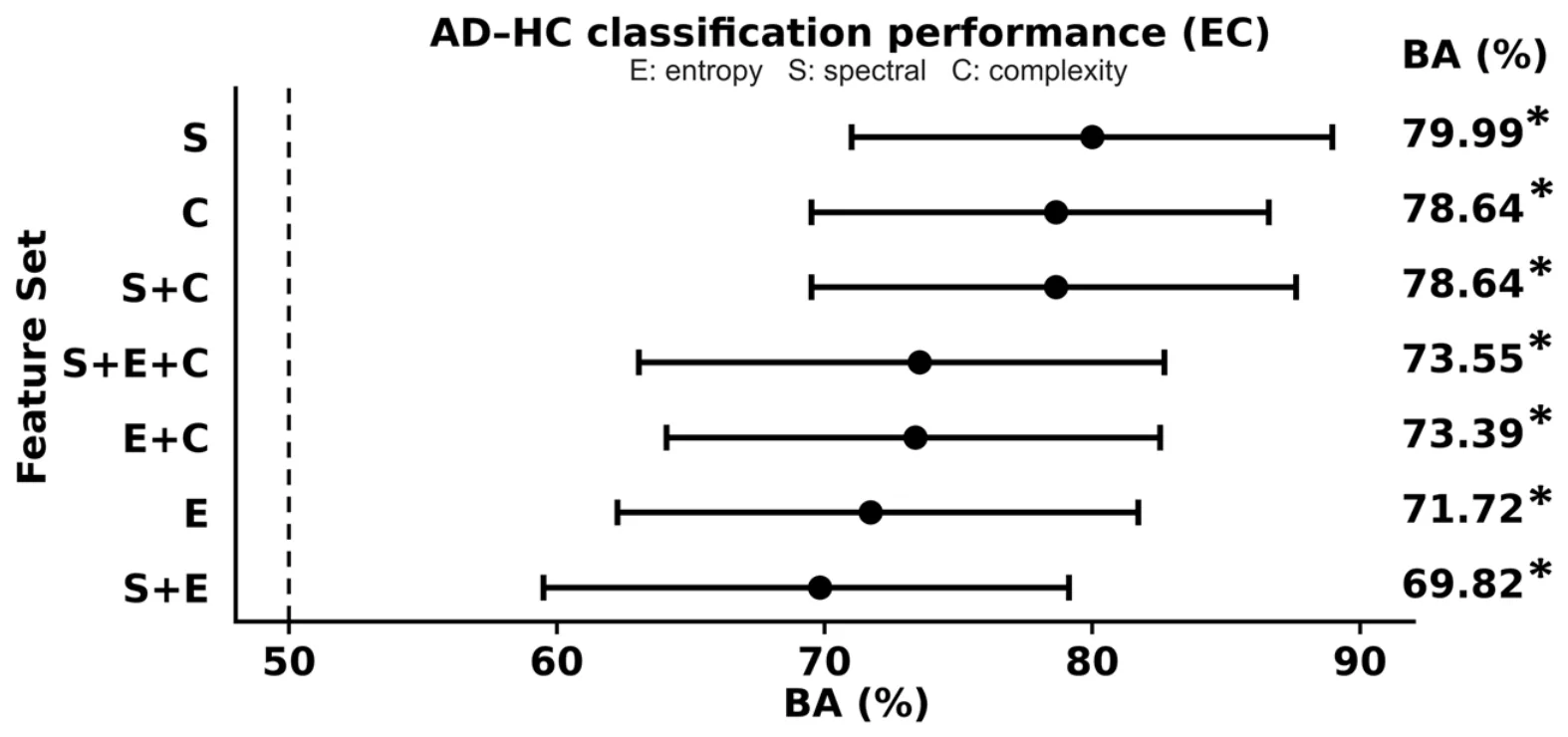
BA across feature configurations for the AD-HC comparison under EC. Points represent observed BA, with horizontal bars indicating 95% CIs; * denotes above-chance performance by permutation testing after FDR correction (*q* < 0.05). The dashed vertical line at BA (50%) indicates chance-level performance for binary classification.

In the AD-HC comparison under the EC condition, the spectral feature set achieved the highest observed BA at 79.99% (95% CI: 71.01–88.96; Figure 3). BA values were similar using both the complexity feature set and the combined spectral–complexity configuration (see Figure 3). Combined feature configurations did not improve performance over individual feature families. Detailed results for all classification metrics other than BA across the seven configurations are provided in the Supplementary Materials (Supplementary Table S2).

For the spectral configuration with the highest BA, the sensitivity and specificity were 76.19% and 83.78%, respectively. The AUC was 0.87, the F1-score was 80.00%, and the precision was 84.21% (Table 3). The corresponding confusion matrix indicated that 32 out of 42 AD participants and 31 out of 37 HC participants were correctly classified (Figure 2), this being in line with the relatively close sensitivity and specificity found for this comparison.

#### 3.2.2. AD vs. MCI

**Figure 4:**
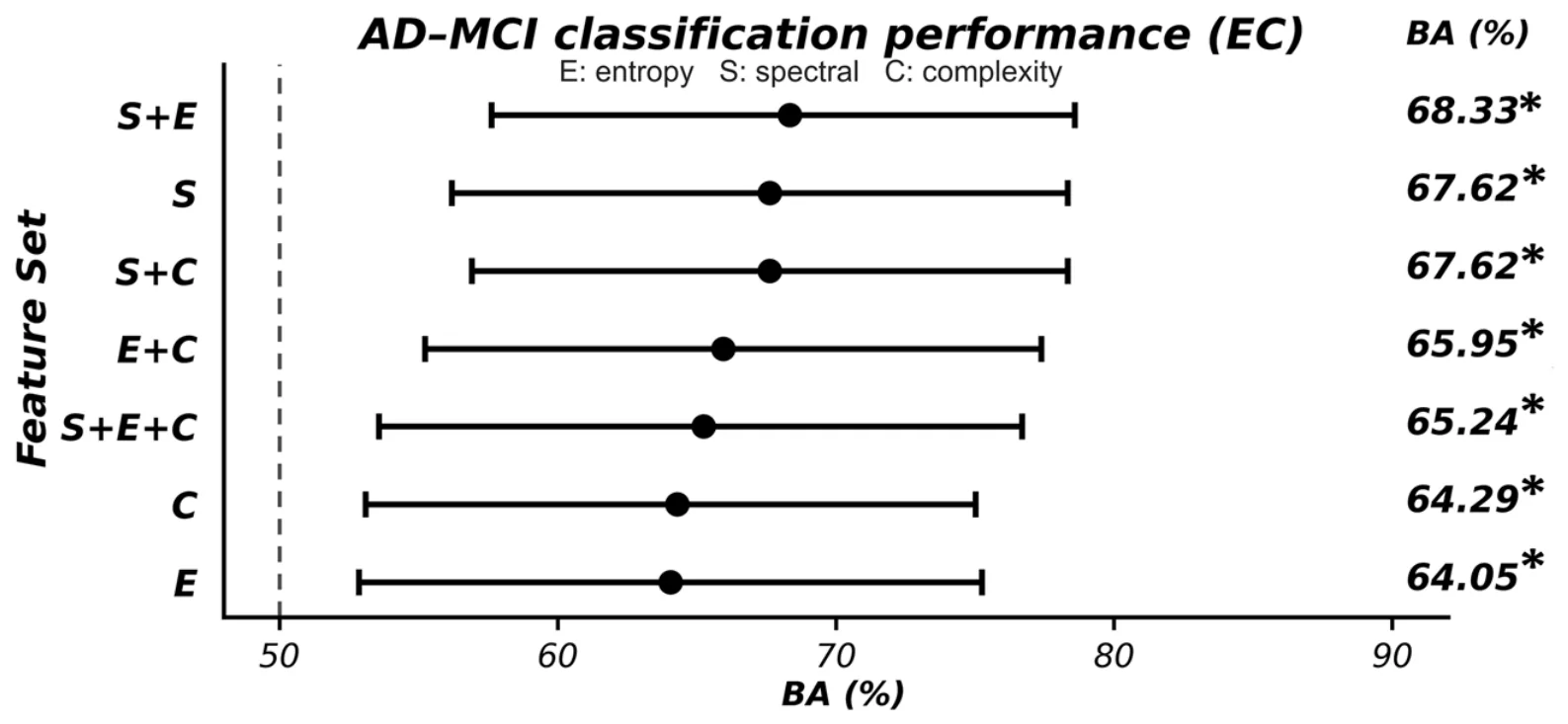
BA across feature configurations for the AD-MCI comparison under EC. Points represent observed BA, with horizontal bars indicating 95% CIs; * denotes above-chance performance by permutation testing after FDR correction (*q* < 0.05). The dashed vertical line at BA (50%) indicates chance-level performance for binary classification.

Distinguishing AD from MCI proved more challenging than differentiating AD from HC. The highest BA was obtained with the spectral–entropy combination at 68.33% (95% CI: 57.61–78.58; Figure 4), followed closely by spectral features alone and the spectral-complexity combination, both at 67.62%. However, the differences between these configurations were small, and their confidence intervals showed substantial overlap (see Figure 4). Notably, spectral features were present in all three top-performing configurations, whereas adding entropy provided little improvement over spectral features alone. These results suggest that spectral information was the primary contributor to classification performance, with limited evidence of additional benefit from feature fusion.

The classification performance of the spectral–entropy configuration was characterised by a marked difference between sensitivity and specificity. Sensitivity was 83.33%, while specificity was 53.33%, indicating substantially better identification of AD compared to MCI participants. Specifically, 35 out of 42 AD participants were correctly classified, whereas only 16 out of 30 MCI participants were accurately classified (Figure 2). The corresponding AUC was 0.66, with an F1-score of 76.92% and a precision of 71.43% (Table 3).

#### 3.2.3. MCI vs. HC

**Figure 5:**
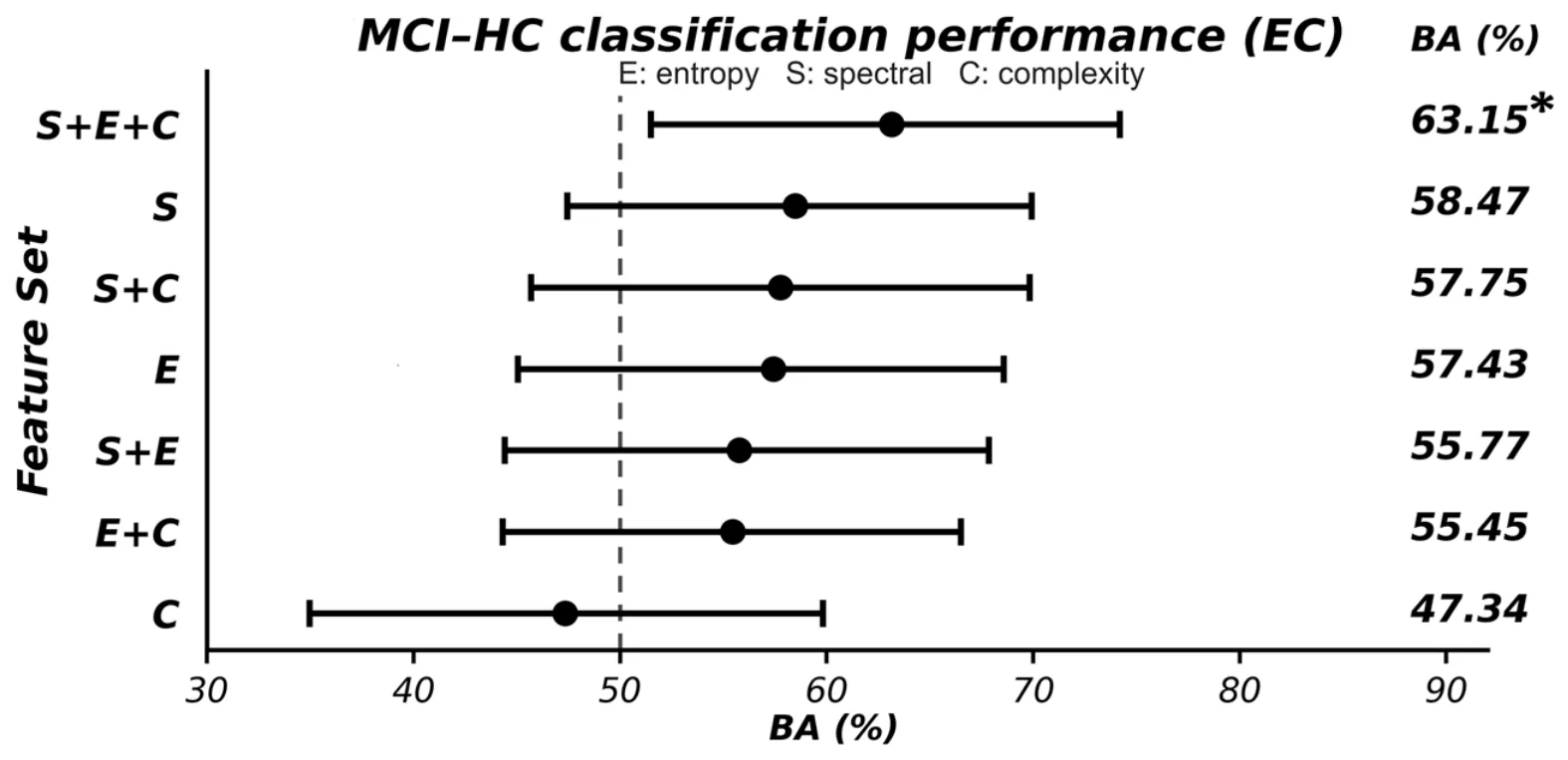
BA across feature configurations for the MCI-HC comparison under EC. Points represent observed BA, with horizontal bars indicating 95% CIs; * denotes above-chance performance by permutation testing after FDR correction (*q* < 0.05). The dashed vertical line at BA (50%) indicates chance-level performance for binary classification.

The MCI-HC classification task was the most challenging among the three EC classification tasks. As shown in Figure 5, the combination of spectral, entropy, and complexity features resulted in the highest BA, at 63.15%. More importantly, permutation testing showed that the combination of spectral, entropy, and complexity features was the only MCI-HC configuration under the EC condition that performed significantly above chance after FDR correction (*q* = 0.0374).

In contrast, the remaining six feature configurations did not significantly exceed chance after FDR correction. Thus, although the complete feature combination showed modest above-chance discrimination, the overall EC MCI-HC findings indicate limited and relatively unstable separability across feature configurations.

For the spectral–entropy–complexity configuration, sensitivity was 53.33% and specificity was 72.97%, indicating a higher correct classification rate for HC than for MCI participants. The AUC was 0.64, with an F1-score of 57.14% and a precision of 61.54%.

### 3.3. Eyes-Open Classification Performance

**Figure 6:**
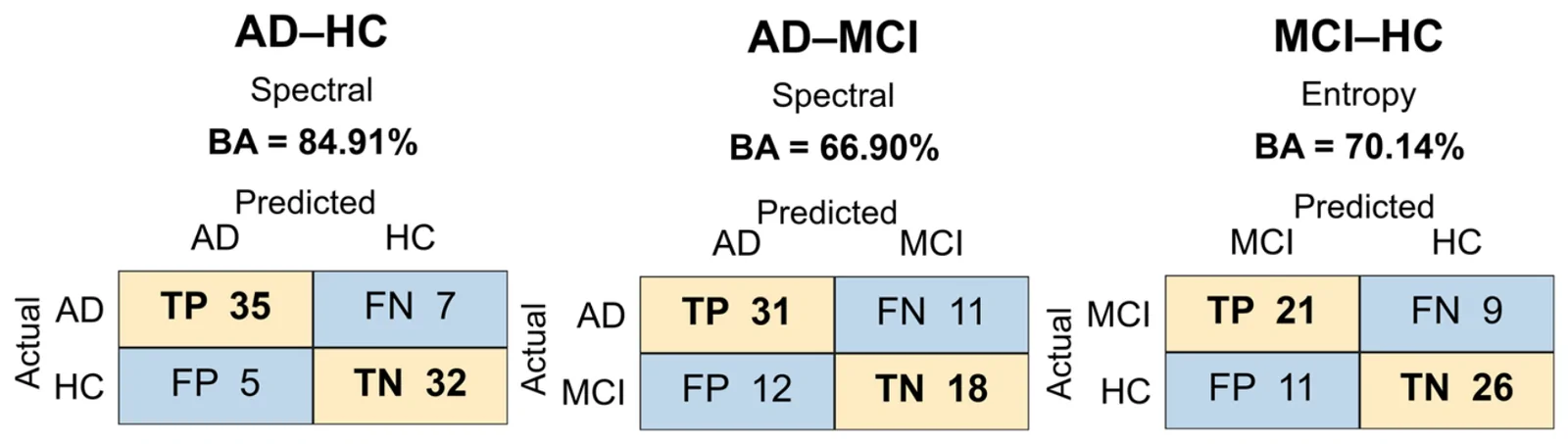
Confusion matrices for the highest BA observed in each diagnostic comparison under the EO condition. Rows indicate the actual class, and columns the predicted class.

Under the EO condition, classification performance varied across the three diagnostic comparisons. The highest observed BA was obtained for AD-HC (84.91%), followed by MCI-HC (70.14%) and AD-MCI (66.90%). The feature configuration associated with the highest BA also differed across comparisons: spectral features alone yielded the highest BA for both AD-HC and AD-MCI, whereas entropy features alone yielded the highest BA for MCI-HC. Notably, in contrast to the EC condition, the highest observed BA for all three EO comparisons was obtained using a single feature family rather than a combined feature configuration. Figure 6 presents the associated confusion matrices, Table 4 summarises the corresponding performance metrics, and complete results across all seven feature configurations are provided in the Supplementary Materials (Supplementary Table S3).

#### 3.3.1. AD vs. HC

**Figure 7:**
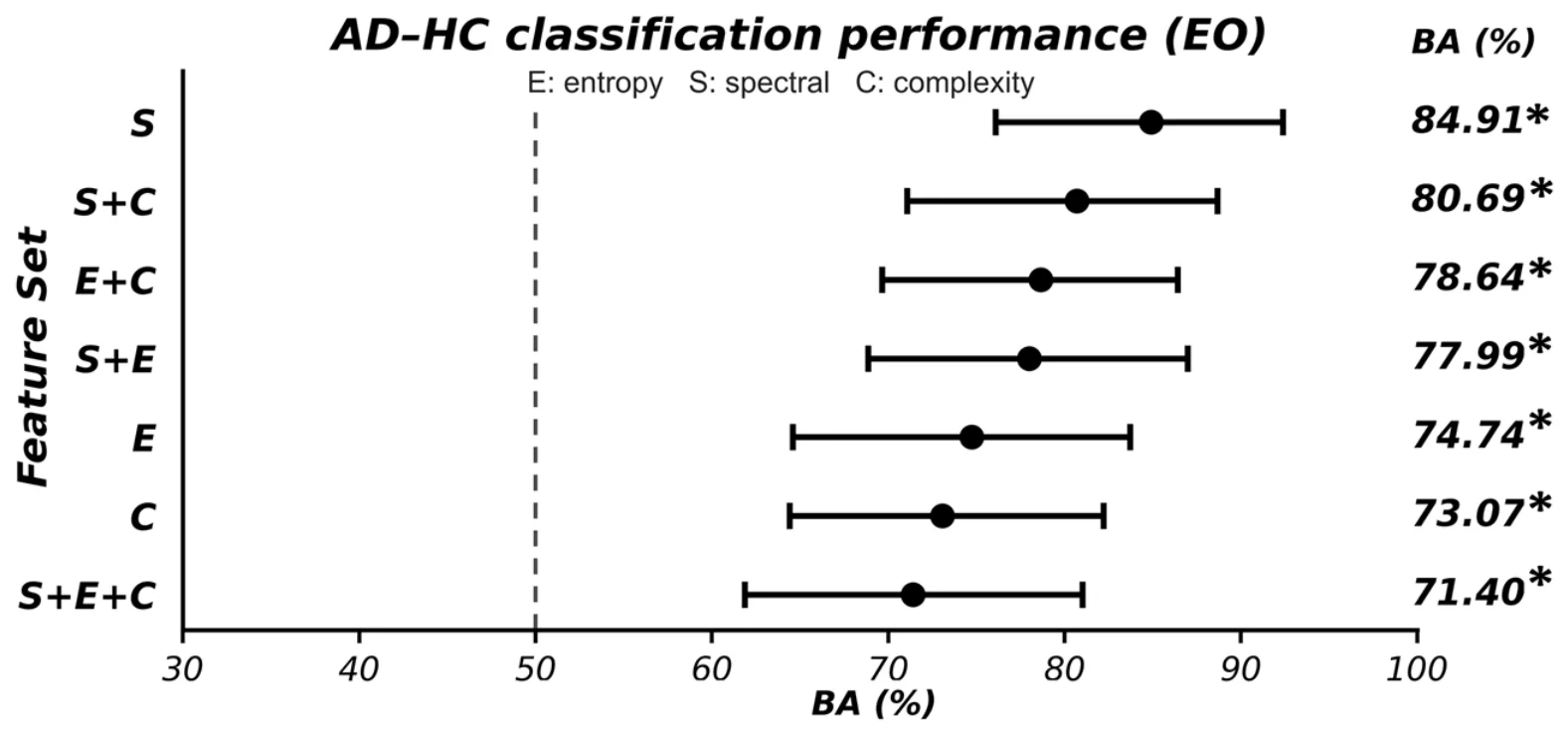
BA across feature configurations for the AD-HC comparison under EO. Points represent observed BA, with horizontal bars indicating 95% CIs; * denotes above-chance performance by permutation testing after FDR correction (*q* < 0.05). The dashed vertical line at BA (50%) indicates chance-level performance for binary classification.

For the AD-HC comparison, the highest classification performance under the EO condition was obtained with the spectral feature set, which achieved a BA of 84.91% (Figure 7). Sensitivity was 83.33% and specificity was 86.49%, indicating similarly strong classification performance for both groups. The model also achieved an AUC of 0.94, together with an F1-score of 85.37% and a precision of 87.50%.

All remaining feature configurations yielded lower BA values, indicating that combining spectral features with entropy or complexity did not improve performance beyond the spectral-only model. The corresponding confusion matrix showed correct classification of 35 of 42 AD and 32 of 37 HC participants.

#### 3.3.2. AD vs. MCI

For the AD-MCI comparison, the highest classification performance under the EO condition was obtained with the spectral feature set, with a BA of 66.90%. Sensitivity was 73.81%, whereas specificity was 60.00%, indicating better identification of AD than MCI participants. The model achieved an AUC of 0.73, with an F1-score of 72.94% and precision of 72.09%. Overall, AD-MCI classification performance was worse than that observed for AD-HC.

**Figure 8:**
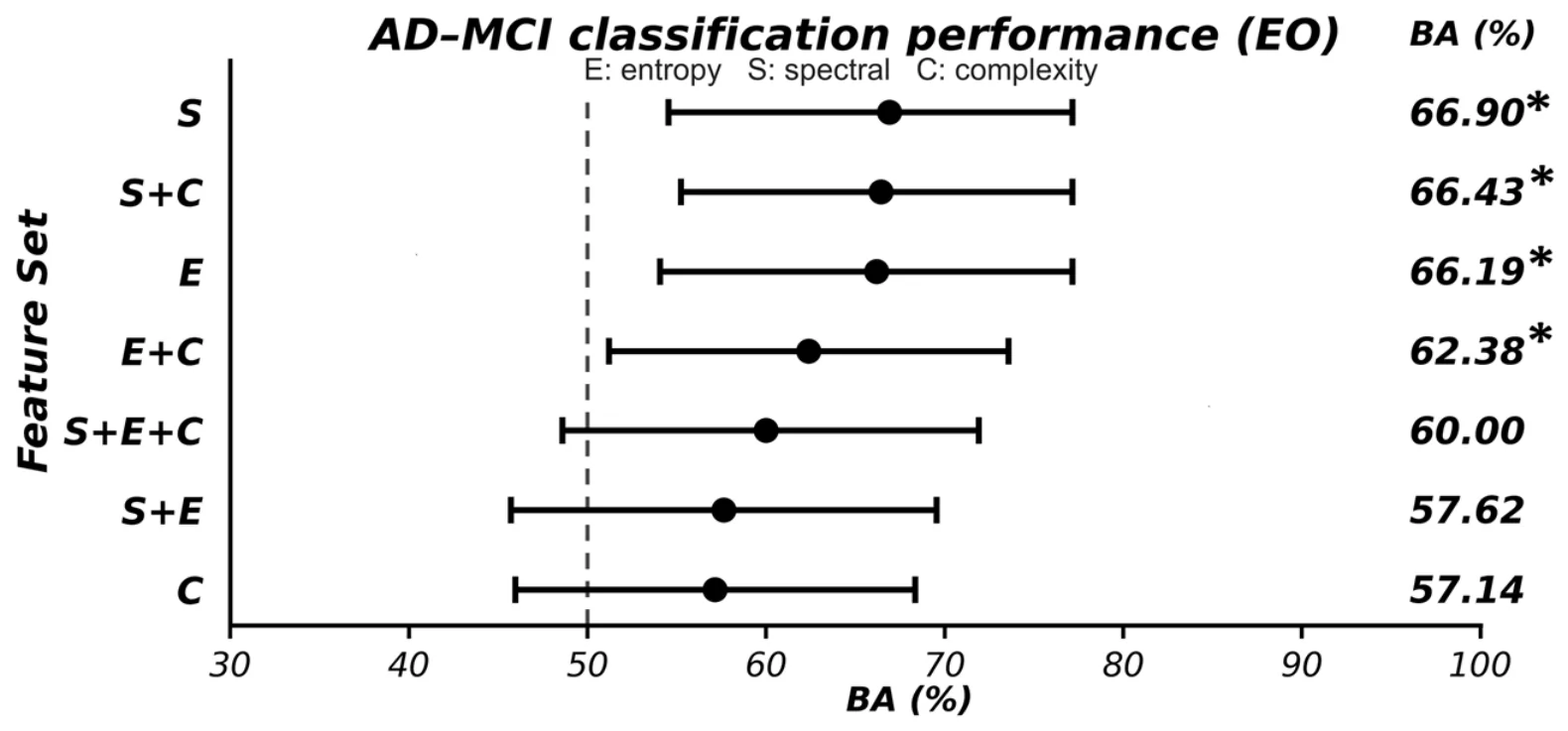
BA across feature configurations for the AD-MCI comparison under EO. Points represent observed BA, with horizontal bars indicating 95% CIs; * denotes above-chance performance by permutation testing after FDR correction (*q* < 0.05). The dashed vertical line at BA (50%) indicates chance-level performance for binary classification.

As shown in Figure 8, none of the combined feature configurations improved BA beyond the spectral-only model. The corresponding confusion matrix showed correct classification of 31 of 42 AD and 18 of 30 MCI participants.

#### 3.3.3. MCI vs. HC

**Figure 9:**
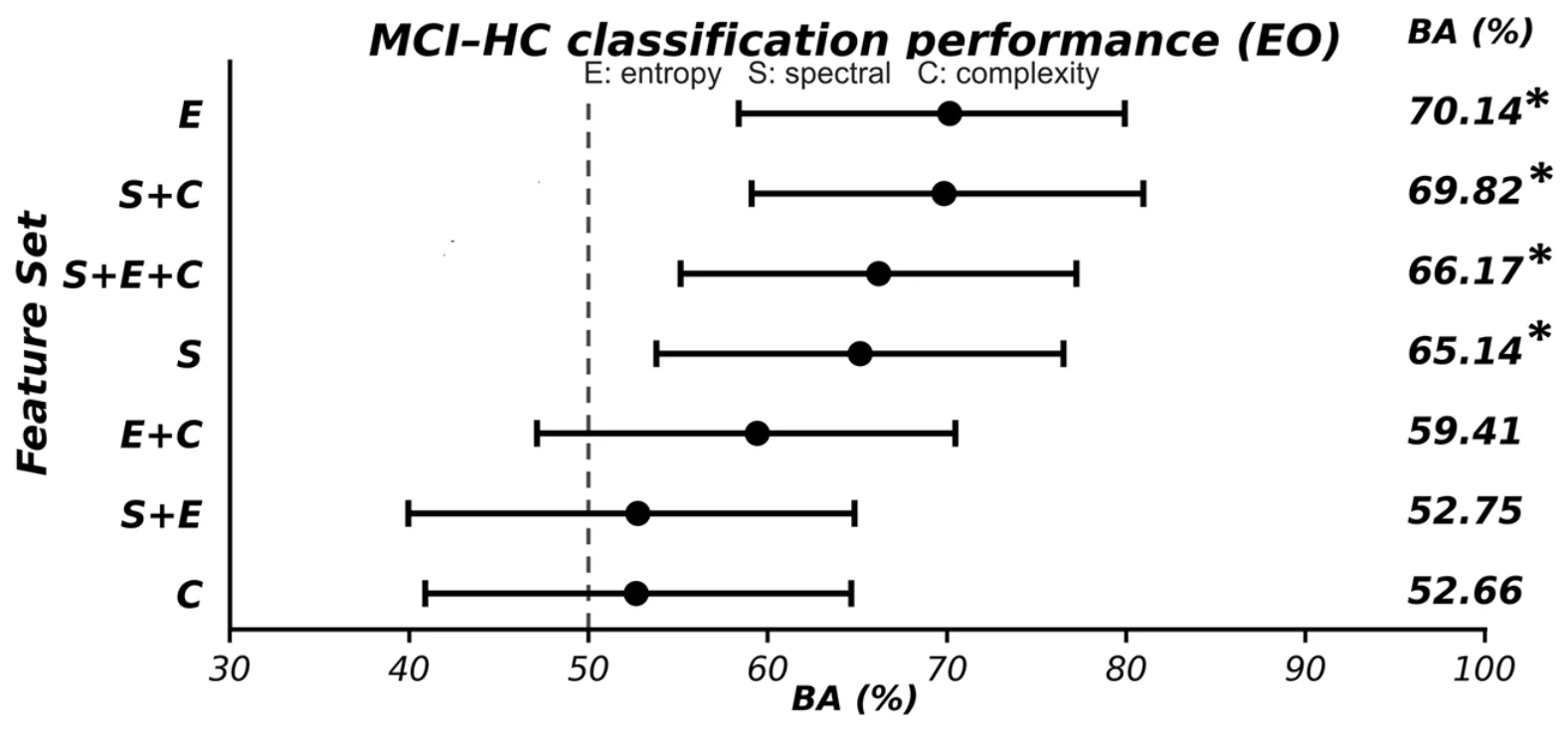
BA across feature configurations for the MCI-HC comparison under EO. Points represent observed BA, with horizontal bars indicating 95% CIs; * denotes above-chance performance by permutation testing after FDR correction (*q* < 0.05). The dashed vertical line at BA (50%) indicates chance-level performance for binary classification.

For the MCI-HC comparison under the EO condition, the highest classification performance was obtained with the entropy feature set (BA of 70.14%; see Figure 9). Sensitivity and specificity were 70.00% and 70.27%, respectively. This BA was significantly above chance in the permutation analysis (*q* = 0.0031). The model achieved an AUC of 0.71, with an F1-score of 67.74% and precision of 65.62%.

Permutation testing further showed that spectral features, the spectral–complexity combination, and the complete spectral–entropy–complexity configuration also performed significantly above chance, whereas complexity, entropy–complexity, and spectral–entropy configurations did not survive FDR correction. Although the entropy set alone yielded the highest BA, combining entropy with spectral and/or complexity features yielded lower BA values. Overall, MCI-HC discrimination was modest, with performance numerically higher than AD-MCI but lower than AD-HC.

### 3.4. Comparison of Feature Families and Feature Fusion

As summarised in Table 5, spectral features demonstrated the most consistent classification performance across diagnostic comparisons in both EC and EO conditions. For all three EC comparisons, the highest-performing configuration included spectral features. Under EO, spectral features alone achieved the highest BA for both AD-HC and AD-MCI. In contrast, entropy features exhibited the strongest relative performance for MCI-HC under EO, where entropy features alone produced the highest BA. Complexity features alone did not achieve the highest performance in any comparison.

The advantage of feature fusion was dependent on both the specific classification task and recording condition, rather than being systematic. Under the EC condition, combined feature configurations yielded the highest observed BA for AD-MCI and MCI-HC, although the improvement for AD-MCI compared to the best single-feature configuration was minimal. On the other hand, under the EO condition, the highest BA for all three diagnostic comparisons was achieved using a single feature family. Furthermore, adding entropy and/or complexity to spectral features did not enhance AD-HC or AD-MCI performance. For MCI-HC, combining entropy with other feature families reduced BA compared to entropy alone.

Overall, combining multiple EEG feature families did not consistently improve classification performance, and the benefit of feature fusion varied across diagnostic comparisons and recording conditions.

### 3.5. Comparison Between EC and EO Conditions

The classification performance under EC and EO conditions was directly compared by pairing the participant-level out-of-fold predictions obtained from the nested LOSO procedure for each feature configuration and diagnostic comparison. The difference in BA between the EO condition and the EC condition was evaluated using a two-sided paired permutation test with 5000 permutations to test the null hypothesis that there was no systematic difference in classification performance between the two conditions. For each permutation, the condition labels were permuted within participants, BA was recalculated separately for the EO and EC conditions, and the difference between the resulting BA values was recomputed. Benjamini–Hochberg false-discovery rate (FDR) correction was subsequently applied across the 21 EC–EO comparisons.

**Figure 10:**
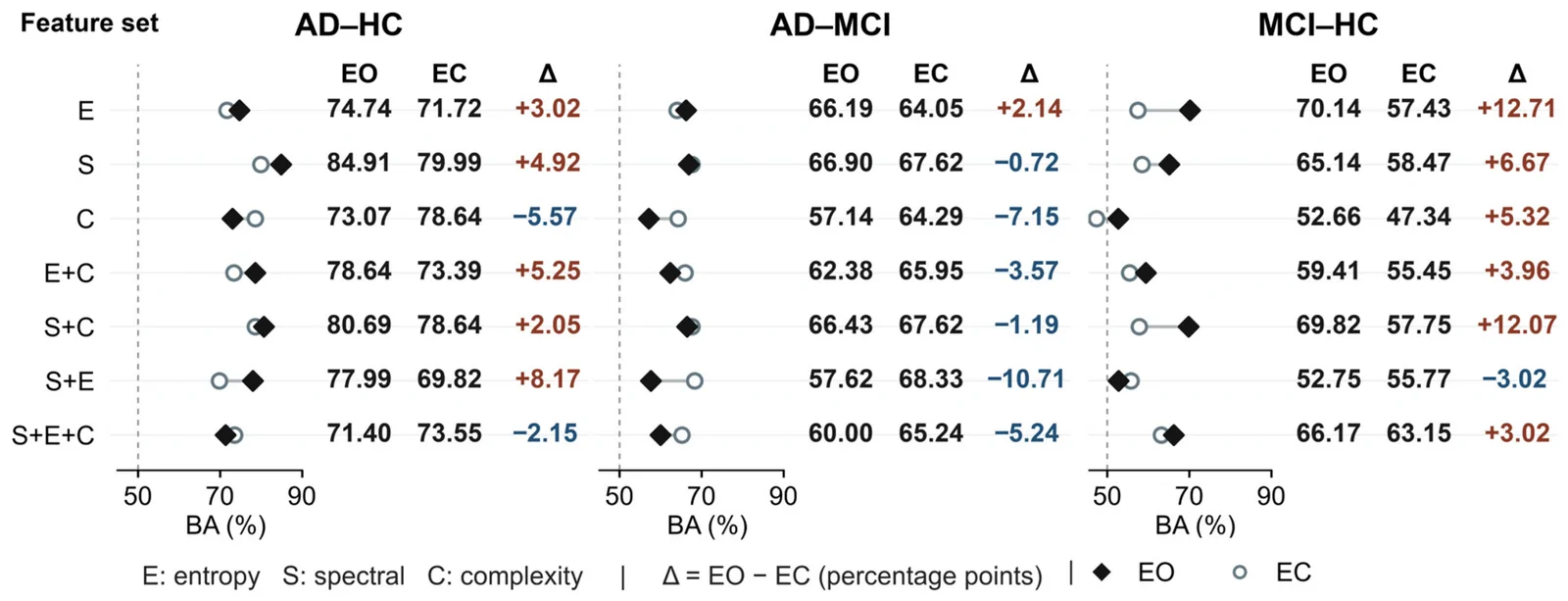
Comparison of BA between EC and EO conditions across feature configurations. Δ represents the difference between EO and EC (EO − EC) in percentage points. Positive Δ values (EO > EC) are shown in red, whereas negative Δ values (EO < EC) are shown in blue.

The magnitude and direction of differences between EC and EO varied by subject group and feature configuration (Figure 10). In AD-HC, most feature configurations showed a numerically higher BA in the EO condition, with the largest increase being for the spectral and entropy combination (+8.17%). In contrast, AD-MCI generally showed small or negative changes across most configurations, with the largest decrease for the spectral and entropy features (−10.71%). The largest positive differences between EC and EO occurred in MCI-HC, especially for entropy (+12.71%; from 57.43% to 70.14%) and for the spectral–complexity features (+12.07%).

None of the 21 EC-EO comparisons achieved statistical significance after paired permutation testing and FDR correction. Although classification performance differed numerically across conditions, these differences were not statistically supported as systematic differences between the EC and the EO conditions. Specifically, the higher performance observed in several MCI-HC configurations in the EO condition should be regarded as a numerical trend rather than evidence of a significant condition effect.

### 3.6. Feature Reduction and Electrode Selection Stability

The initial feature pool consisted of 285 spectral, 305 entropy, and 152 complexity features. After the two-stage feature-selection procedure, significantly smaller feature sets were retained across LOSO folds, as summarised in Table 6. Stage 1 markedly reduced all three feature domains, while Stage 2 further restricted the feature space to relatively compact subsets.

To examine the spatial distribution of feature selection, we evaluated how often features associated with each electrode were selected across all LOSO folds. For each diagnostic comparison, recording condition, and feature domain, the number of selections associated with each electrode was divided by the total number of electrode-associated feature selections and expressed as a percentage. Electrodes were then ranked by these percentages, and the smallest set accounting for at least 50% of all selections was defined as the high-contribution electrode set (Table 7). The corresponding electrode-level distributions are provided in the Supplementary Materials (Supplementary Figures S1–S3 for EC and Figures S4–S6 for EO).

In the AD-HC comparison, frequently selected electrodes consistently reflected contributions from temporal and temporo-parietal regions across both recording conditions. In the EC condition, the spectral features mainly included temporal electrodes T3, T4, and T6, as well as Fz. Complexity features showed a similar pattern, with T3 and P3 as the main contributors. In the EO condition, temporal involvement continued: T4 was part of the spectral features, T4 and T6 contributed to the entropy features, and T4 and T5 were included in the complexity features. Although the specific electrodes varied between the EC and EO conditions, the selected features were associated with electrodes located over temporal and adjacent regions that contributed to distinguishing AD from HC. The AD-MCI comparison depended more on the recording condition. With EC, the main sets showed more activity in the frontal, central, and middle areas. Spectral features were strongest at Fz, C3, and C4, while the entropy set included electrodes mostly in the frontal region, including F3, F4, F7, and Fz. Complexity features were stronger in the central and parietal locations. Under the EO condition, the spatial distribution shifted towards temporal and posterior regions. T4, T5, P3, and Pz were part of the main group for the spectral features, while T3, T5, Pz, and O2 were included in the main group for the entropy features. Posterior electrodes, particularly Pz and O2, were also present among the complexity features. Overall, AD-MCI showed the largest change in the spatial distribution of frequently selected electrodes between the EC and EO conditions.

For MCI-HC, frontal and central contributions were more apparent, especially under EO. Under the EC condition, the spectral features showed a distributed pattern involving parietal, temporal, central, frontotemporal, and occipital electrodes (P3, T3, C3, F8, and O2). In contrast, the entropy and complexity features included several frontal electrodes, such as F3, Fz, Fp1, Fp2, and F7. This frontal tendency was more pronounced under the EO condition, where four of the six spectral frequently selected electrodes were frontal or frontotemporal (F3, F8, Fp2, and F4). Frontal and central electrodes also remained strongly represented within the entropy and complexity features, with recurrent contributions from F3, F4, F7, C4, and T4.

In conclusion, frequently selected electrodes indicated that the spatial organisation of selected features differed by both diagnostic comparison and recording condition. AD-HC showed a relatively consistent temporal and temporo-parietal pattern across both EC and EO, whereas AD-MCI demonstrated the most pronounced condition-related spatial redistribution, with relatively greater frontal/central representation under EC and greater temporal/posterior representation under EO. MCI-HC showed stronger frontal and frontal–central representation, particularly under EO. Across all comparisons, occipital electrodes were only occasionally represented among the frequently selected electrodes.

## 4. Discussion

This study systematically evaluated spectral, entropy-based, and complexity-based resting-state EEG features, both individually and in combination, for classifying AD-HC, AD-MCI, and MCI-HC under EC and EO conditions. The main findings are as follows: First, spectral features were the most consistently useful features across group comparisons, resulting in the highest BA for AD-HC in both recording conditions and for AD-MCI in EO, and also being part of the best EC feature setups for AD-MCI and MCI-HC. Second, combining features did not generally improve performance. While the spectral–entropy setup reached the highest BA for AD-MCI in EC, it only slightly improved on spectral features alone. Similarly, the spectral–entropy–complexity combination resulted in the highest BA for MCI-HC in the EC condition and was the only configuration that remained significantly above chance after FDR correction. Third, although there were numerical differences between BA in the EC condition and the EO condition, especially for MCI-HC, none of the 21 paired EC-EO comparisons remained significant after permutation testing and FDR correction. The results therefore suggest that EEG feature utility depends on the diagnostic group comparison and recording condition, rather than supporting a uniform advantage of either one recording condition or a larger fused feature space.

### 4.1. Spatial Interpretation

The electrode-selection analysis provides a spatial perspective on the classification results; however, these findings should be interpreted as evidence of selection recurrence rather than as direct evidence of the magnitude of physiological abnormalities. We defined the frequently selected electrodes as those collectively accounting for at least 50% of electrode-associated selections across the outer LOSO folds. Accordingly, recurrent selection indicates that these electrode locations were consistently informative for classification across the outer folds.

For AD-HC classification, temporal contributions were relatively consistent across recording conditions. Under EC, the spectral features included T3, T4, and T6, whereas the complexity features were dominated by T3 and P3. Temporal involvement was also evident under EO: T4 and F8 were represented in the spectral features; T4, T6, and F7 in the entropy features; and T4, T5, and F8 in the complexity features. This distribution is compatible with established AD-related spectral abnormalities in temporal regions. Previous work has reported higher relative temporal theta power and altered beta2 activity over frontal and temporal regions in AD compared with HC [41]. Consistent with these findings, another study reported increased theta power over temporal–parietal and occipital regions, together with reduced beta power at temporal and frontal sites, including T3 and T4 [42]. In addition, temporal theta relative power has been significantly associated with MMSE scores in patients with AD [43]. Although MMSE associations were not examined directly in the present study, the recurrent temporal contribution observed here is consistent with the broader body of literature identifying temporal EEG abnormalities as a prominent feature of AD. The present analysis extends this observation by showing that the spatial pattern persisted across EC and EO conditions within a nested, subject-level classification framework.

The entropy and complexity selections are also consistent with previous evidence that AD-related neurodegeneration affects signal irregularity and temporal organisation, particularly in temporal and parietal regions. In the present analysis, P3 was recurrently selected across both EC and EO conditions, alongside temporal electrodes, including T3, T4, T5, and T6; frontal and central sites were also represented. Previous studies have reported reduced approximate entropy at parietal and occipital electrodes in AD [16], as well as reductions in approximate, sample, and fuzzy entropy across temporal and parietal sites, including T3, T4, C3, P3, and P4 [44]. Similarly, lower signal complexity has been reported at temporal, parietal, and occipital sites using AMI rate [16], LZC [45], and HFD [46,47]. These findings provide a plausible contextual basis for the recurrent temporo-parietal involvement observed in the present AD-HC models. However, because the current analysis reflects feature-selection frequency rather than group-wise feature values, these results should be interpreted as contextual support rather than direct evidence of reduced entropy or complexity in the present sample.

In contrast to the relatively consistent temporal contribution observed in the AD-HC comparison, the AD-MCI results showed greater condition-dependent variation in the spatial distribution of selected features. Under the EC condition, the entropy features exhibited prominent frontal involvement, including F4, Fz, F3, and F7, consistent with previous reports of altered EEG entropy and complexity measures in frontal regions across the MCI-AD continuum [48]. Under the EO condition, the entropy features included a greater proportion of temporal and posterior electrodes, including T5, T3, Pz, and O2. Frequently selected electrodes for complexity features showed a broader spatial distribution under EC, spanning central, parietal, temporal, and frontal sites, whereas under EO, they were mainly distributed across posterior, frontal, and central regions. Thus, while the comparison between AD and HC demonstrated a consistent temporo-parietal pattern across recording conditions, the comparison between AD and MCI exhibited greater variability in the topographic distribution of selected features between EC and EO conditions. Because the direct EC-EO classification comparisons did not show significant differences, these variations should nevertheless be interpreted as descriptive tendencies in feature selection rather than evidence of condition-specific effects.

For MCI-HC classification, the most notable pattern was the recurrence of frontal–central involvement across both recording conditions. Under EC, the entropy and complexity features included several frontal and central electrodes, including F3, Fz, Fp1, Fp2, F7, C4, and Cz. Under EO, the spectral features exhibited a more pronounced frontal and fronto-temporal distribution, including F3, F4, F8, Fp2, T3, and T4, while frontal and central sites remained represented in the entropy and complexity features. This recurrent frontal–central contribution is consistent with previous reports of frontal and central EEG alterations in MCI-HC discrimination [49], as well as with Hjorth-parameter findings showing significant differences at the frontal electrodes F3 and Fz [50].

More generally, EC and EO are known to influence the organisation of resting-state EEG activity. Eye closure is typically associated with increased alpha activity and reduced sensory processing, whereas eye opening suppresses alpha rhythms and may increase cortical engagement associated with visual input [51,52,53]. These condition-dependent physiological differences provide a plausible context for the observed variation in the composition and spatial distribution of the multielectrode feature sets selected under EC and EO.

### 4.2. AD-HC

Among the group comparisons, AD-HC discrimination showed the highest overall classification performance under both EC and EO conditions. The best BA results were obtained with spectral features under both EC and EO, but performance was higher under EO than EC: BA increased from 79.99% to 84.91%, sensitivity from 76.19% to 83.33%, and specificity from 83.78% to 86.49%. AUC showed the same pattern, increasing from 0.87 under EC to 0.94 under EO. Importantly, adding entropy or complexity features did not improve BA beyond the spectral-only model in either condition. Thus, across both recording conditions, spectral information made the most robust and consistent contribution to AD-HC discrimination, with EO yielding the stronger descriptive performance profile.

This finding is particularly relevant given the strong emphasis on EC recordings in the resting–awake EEG literature. Of 98 studies, 85 used only EEG recordings under the EC condition, while only 13 included both EC and EO, and just three of the 13 studies analysed the two conditions separately [2]. Although previous classification studies have predominantly focused on EC recordings, the present findings indicate that EO recordings may offer comparable, and potentially complementary, discriminatory information for AD-HC classification, supporting their further investigation in future studies.

The relatively stable background rhythms observed during resting, EC conditions might be expected to support discrimination between AD and HC [52]. In particular, the dominance of occipital alpha activity in the EC condition may facilitate a clearer demonstration of disease-related differences in power distribution between AD and HC groups using spectral measures. Contrary to this expectation, however, the spectral feature set achieved improved BA for AD-HC classification under EO in the present study. Visual input and the associated increase in alertness can alter the organisation of resting EEG and attenuate the dominant alpha rhythm [53], potentially resulting in a more dynamically reorganised cortical state. This altered state may have made disease-related differences in neural reactivity and signal organisation more detectable in individuals with AD, thereby improving group separability. Thus, although the more stable spectral organisation of EC might theoretically favour discrimination, the greater dynamic variation associated with EO may have enhanced AD-related abnormalities and contributed to the higher observed classification performance. This interpretation remains provisional, however, because the observed EC–EO differences were not supported by significant paired comparisons after correction for multiple testing.

For AD-HC discrimination, previous spectral-based studies have reported a wide range of performance estimates. One study using PSD features achieved 95.6% accuracy in a data set comprising 19 AD and 17 HC participants [54], whereas another, based on 36 AD and 29 HC participants, reported an accuracy of 77.01% using spectral features [55]. Similarly, Ref. [56] evaluated spectral alpha-peak parameters in 22 AD and 12 HC participants and achieved 91.8% accuracy with an SVM. Ref. [57] reported an AUC of 0.92 for AD-HC discrimination using the alpha-to-theta ratio in a cohort of 50 AD and 57 HC participants.

However, direct comparison with these values should be made cautiously, because studies differ in sample size, validation design, feature selection, preprocessing, recording protocol, and the performance metric reported. Importantly, the choice of validation strategy can substantially affect reported accuracy. For example, in an EEG-based schizophrenia study, accuracy decreased from 98% with 10-fold cross-validation to 70% with LOSO, a 28-percentage-point drop [58]. An even more striking discrepancy appeared in their subject-wise label-corruption experiment: k-fold cross-validation still yielded 98% accuracy, whereas LOSO accuracy dropped to 30%, a difference of 68 percentage points. These findings illustrate how conventional sample-level k-fold validation can produce highly optimistic estimates when subject-specific information is shared between the training and test sets. In the present study, we used an even more stringent nested LOSO framework, in which each subject was held out entirely in the outer loop, while feature selection and hyperparameter tuning were performed exclusively within the corresponding training data. Consequently, the comparatively lower accuracies obtained here may reflect the greater rigour of the validation procedure and a more realistic estimate of generalisation to unseen subjects, rather than inherently poorer predictive capability.

Beyond spectral features, entropy- and complexity-based representations also provided meaningful, though more modest, AD-HC discrimination. Under EC, complexity features achieved a BA of 78.64%, compared with 71.72% for entropy features. Under EO, the corresponding BA values were 73.07% and 74.74%, respectively. Although these non-linear descriptors did not yield the strongest overall performance, they still captured disease-related information supporting moderate discrimination. These findings should be interpreted within the validation framework and reported accuracy metric, rather than as evidence that entropy or complexity measures independently reflect a specific physiological abnormality.

The entropy-based results can be interpreted alongside previous AD-HC studies, several of which have reported higher classification performance. Multiscale entropy yielded ROC-based accuracy estimates ranging from 81.82% to 90.91% across electrodes in a small cohort of 11 AD and 11 HC participants [59], whereas a larger study of 51 AD and 50 HC participants reported 79.1% accuracy using multiscale entropy with QDA [60]. Similarly, fuzzy entropy achieved 86.36% accuracy in a cohort of 11 AD and 11 HC participants [17], while sample entropy has been reported to yield 85% accuracy [44]. Transfer entropy produced accuracies of 87.5% under EC and 93.8% under EO in a study involving 17 AD and 15 HC participants [61]. By comparison, entropy-based performance in the present study was more modest, reaching BA values of 71.72% under EC and 74.74% under EO.

Overall, the results indicate that spectral features provided the principal EEG information supporting AD-HC classification in the present cohort, whereas non-linear measures captured additional but comparatively weaker differences in signal organisation. Within the rigorous nested, subject-level validation framework, this pattern was observed under both EC and EO conditions, supporting the robustness of spectral abnormalities as discriminative descriptors of AD-related EEG alterations. This interpretation concerns classification utility rather than establishing spectral features as specific physiological biomarkers of AD.

### 4.3. AD-MCI

AD-MCI classification was more challenging than AD-HC classification under both recording conditions. Under the EC condition, the spectral–entropy combination achieved the highest BA of 68.33%; however, spectral features alone and the spectral–complexity combination both achieved a very similar BA of 67.62%. The best-performing EC model also showed relatively high sensitivity (83.33%) but low specificity (53.33%), indicating that the classifier identified AD participants more successfully than MCI participants. Given that the addition of entropy improved BA by only 0.71 percentage points over the spectral-only model, this small difference does not provide strong evidence of a meaningful synergistic effect. Instead, the results suggest that spectral information was the main contributor to AD-MCI discrimination under the EC condition. A similar pattern was observed under the EO condition, where the spectral feature set alone produced the highest BA of 66.90%, with a sensitivity of 73.81%, specificity of 60.00%, and an AUC of 0.73.

Overall, these findings indicate that spectral features were consistently important for AD-MCI discrimination, whereas the incremental contribution of entropy was small and was observed only under the EC condition. Previous studies have nevertheless reported relatively high classification performance using spectral- and entropy-based EEG features. For example, a combination of spectral and entropy features achieved an accuracy of 76.47% for distinguishing AD from a combined MCI + HC group [62]. In a study using PSD-based features, an accuracy of 84.62% was reported for direct AD-MCI discrimination in a data set comprising 63 AD and 63 MCI participants [63]. Similarly, another study combining spectral and entropy features reported an accuracy of 90.9% for AD-MCI classification in a smaller cohort of 17 AD and 16 MCI participants [61]. The lower BA performance observed in the present study may, as discussed previously, partly reflect the use of LOSO validation, which evaluates generalisation to entirely unseen participants and can therefore yield more conservative performance estimates.

The difficulty of AD-MCI separation is biologically plausible because MCI may share part of the neurophysiological continuum associated with AD [64]. The limited specificity in both conditions suggests that the selected EEG features did not clearly separate the two groups. Frequently selected electrodes also differed by condition, with frontal and central locations more prominent in EC and temporal and posterior locations in EO. This variation may indicate that EEG features relevant to AD-MCI discrimination are expressed differently under the EC and the EO conditions, rather than forming a stable spatial pattern across both conditions.

### 4.4. MCI-HC

The MCI-HC group remained the most uncertain classification problem. Under the EC condition, the spectral–entropy–complexity combination achieved the highest BA of 63.15% and was the only configuration that remained significantly above chance after FDR correction (*q* = 0.0374). However, this model showed a marked imbalance between sensitivity (53.33%) and specificity (72.97%), indicating that HC participants were identified more accurately than MCI participants. Thus, although the fused feature set produced the highest BA under EC, this result should be interpreted cautiously and does not provide strong evidence that combining all feature families consistently improves MCI-HC discrimination.

In contrast, classification performance was stronger under the EO condition. Entropy features alone achieved the highest BA of 70.14%, with nearly balanced sensitivity (70.00%) and specificity (70.27%), and this performance remained significantly above chance after FDR correction (*q* = 0.0031). Spectral features, the spectral–complexity combination, and the spectral–complexity–entropy combination also remained significant after FDR correction; however, none exceeded the performance of the entropy-only model. These findings suggest that, under the EO condition, entropy-related information provided the strongest discrimination between MCI and HC participants, while the addition of other feature families did not yield further improvement in BA.

The apparent advantage of the EO condition was particularly evident for entropy features, for which BA increased numerically from 57.43% under EC to 70.14% under the EO condition. This represented one of the largest EC–EO differences observed in this study. Nevertheless, the paired permutation analysis indicated that this difference, as well as the other EC–EO differences, did not remain significant after multiple-comparison correction. Therefore, the higher EO performance should be regarded as a numerical trend rather than evidence of a statistically reliable condition effect. More broadly, the substantial variability reported in the MCI classification literature [61] may reflect differences in sample heterogeneity, MCI subtype definitions, recording protocols, feature-selection strategies, and validation procedures.

### 4.5. Feature Fusion and Recording-Condition Effects: Methodological Implications

Across the six comparisons (two eye conditions × three diagnostic comparisons), the results do not support indiscriminate feature fusion. Spectral features were included in every highest-performing configuration under the EC condition and, under the EO condition, achieved the highest BA among the individual feature families for both AD-HC and AD-MCI classification. By contrast, complexity features alone did not constitute the best-performing configuration in any diagnostic comparison under either condition. Feature fusion yielded the highest observed BA only for EC AD-MCI and EC MCI-HC. In the former comparison, however, the improvement over spectral features alone was negligible. In the latter, the spectral–entropy–complexity combination achieved the highest BA and remained significantly above chance after FDR correction, but its relatively low sensitivity compared with specificity indicated an imbalance in the classification of MCI and HC participants. Moreover, under EO, the highest BA for each of the three diagnostic comparisons was obtained using a single feature family rather than a fused feature set. Taken together, these findings suggest that increasing feature diversity does not necessarily provide additional independent diagnostic information. Spectral, entropy, and complexity descriptors may partly capture overlapping manifestations of the same underlying EEG alterations, while the inclusion of weak or redundant variables may increase model variance without producing corresponding gains in generalisation performance.

The two-stage hierarchical feature-selection procedure substantially reduced the initial feature space within each outer LOSO fold, resulting in relatively compact feature subsets for final model training. Such dimensionality reduction is particularly important in the present setting, where hundreds of candidate EEG features were derived from a comparatively modest number of participants. At the same time, variation in both the number of selected features and the electrode identities retained across folds suggests that the discriminative information was distributed across multiple features and spatial locations rather than being represented by a single invariant marker. In this context, the core-electrode analysis provides a complementary perspective to predictive performance by identifying spatial patterns that recurred across folds. Nevertheless, the identification of stable clinically relevant biomarkers would require independent replication not only of classification performance but also of the associated feature-selection and spatial-selection patterns.

The direct comparison between EC and EO conditions provides an additional methodological perspective. Several point estimates suggested potentially meaningful condition-related differences, most notably the higher EO performance observed for several MCI-HC configurations, whereas differences for AD-HC and AD-MCI were generally smaller. However, none of the 21 paired EC–EO comparisons remained significant after FDR correction. Accordingly, the observed numerical differences do not provide sufficient evidence to favour one recording condition over the other on the basis of the present data alone. Rather than selecting EC or EO solely according to the highest observed point estimates, recording condition should be considered part of the broader modelling context and its contribution evaluated prospectively.

### 4.6. Limitations and Future Directions

Several limitations of this study should be considered. First, a single public data set, with a relatively modest age-matched paired subset was used, limiting generalisability to other populations and clinical settings. Although nested LOSO cross-validation reduces optimistic bias within the available sample, it cannot substitute for external validation. The relatively wide confidence intervals, particularly for AD-MCI and MCI-HC classification, further indicate uncertainty around the point estimates. Future studies should therefore evaluate the complete analytical pipeline in larger and independent cohorts.

Second, although age was controlled across groups, several clinically relevant variables were unavailable in the data set, including MMSE score, education, medication status, sex, and disease severity. These factors may contribute to EEG heterogeneity and may be particularly relevant to the more challenging AD-MCI and MCI-HC comparisons.

Third, the electrode-stability analysis quantified the recurrence of electrode-associated feature selection but did not directly estimate the magnitude of group differences for each selected feature. Accordingly, the regional interpretation should be understood as a description of topographic predictive relevance rather than as a spatial map of disease-related physiological alterations. Region-wise averaging may improve interpretability and reduce dimensionality, whereas synchronisation and functional-connectivity measures could provide complementary information about inter-regional network disruption.

Finally, the analysis focused on a fixed set of spectral, entropy-based, and complexity-based descriptors and used a single LightGBM-based feature-selection and classification framework. The extracted entropy and complexity features also depend on methodological-parameter choices, such as embedding dimension, tolerance, time delay, and scale, where applicable. These parameters were not optimised using the present data set, and their sensitivity to alternative settings was not systematically evaluated. Consequently, the comparison should be interpreted as reflecting the performance of the implemented feature configurations under the specified parameter settings rather than as a definitive ranking of spectral, entropy, and complexity approaches.

Alternative classifiers, feature-selection procedures, ensemble approaches, and systematic optimisation of feature-extraction parameters may improve predictive performance, stability, or interpretability. Nevertheless, our findings highlight the potential of resting-state EEG features, particularly spectral measures, for distinguishing among AD, MCI, and HC, while emphasising the importance of considering diagnostic group comparison and recording condition when developing EEG-based classification approaches.

## Figures and Tables

**Table 1:** Summary of participant demographics and a statistical comparison of age across diagnostic groups for the EO/EC conditions.

Data Set	Groups	*n*	Mean Age ± *SD*	Statistical Test
EO/EC	AD	42	66.95 ± 7.62	
	HC	37	67.62 ± 6.95	ANOVA: *F* (2,106) = 0.17, *p* = 0.845, η^2^ = 0.003
	MCI	30	67.93 ± 7.64	

**Table 2:** Summary of extracted spectral, entropy-based, and complexity-based EEG features used for classification.

Feature Set	Subcategory	Extracted Feature Metrics
Complexity features	Complexity metrics	Auto mutual information rate (AMIR) [21]; central tendency measure (CTM) [22]; detrended fluctuation analysis (DFA) [23]; Higuchi Dimension (HFD) [24]; Hjorth complexity (HJ-C) [25]; Hjorth mobility (HJ-M) [25]; Katz Dimension (KFD) [26]; Lempel–Ziv complexity (LZC) [27]
Spectral features	Alpha peak parameters	Bandwidth (BW) [28]; centre frequency (CF) [28]; exponent [28]; offset [28]; power (PW) [28]
	Median frequency (MF)	Theta; alpha; total [29]
	Power ratios	Theta/(alpha + beta); theta/alpha
	Relative power (RP)	Delta; theta; alpha; beta; gamma [30]
Entropy features	Single scale	Attention entropy (AE) [31]; bubble entropy (BubbleEn) [32]; cross-fuzzy entropy (XFuzzyEn) [33]; entropy of entropy (EoE) [34]; fuzzy entropy (FuzzyEn) [35]; range entropy (RangeEn) [36]; wavelet entropy (WE) [37]
	Multiscale, multivariate	Multiscale sample entropy (MSE) [38]; multivariate multiscale sample entropy (mvMSE) [39]

**Table 3:** Classification metrics for the feature configuration yielding the highest BA in each diagnostic comparison under the EC condition.

Comparison	Features	BA (%)	Sensitivity (%)	Specificity (%)	AUC	F1-Score (%)	Precision (%)
AD-HC	S	79.99 [71.01–88.96]	76.19 [61.90–88.10]	83.78 [70.27–94.59]	0.87 [0.78–0.95]	80.00 [70.13–89.16]	84.21 [74.42–94.29]
AD-MCI	E + S	68.33 [57.61–78.58]	83.33 [71.43–92.86]	53.33 [36.67–70.00]	0.66 [0.51–0.79]	76.92 [68.68–84.44]	71.43 [63.64–80.01]
MCI-HC	C + E + S	63.15 [51.48–74.19]	53.33 [36.67–70.00]	72.97 [56.76–86.49]	0.64 [0.51–0.76]	57.14 [42.11–70.77]	61.54 [46.67–76.92]

Note. The 95% CIs were estimated using 1000 stratified bootstrap resamples of the outer-loop LOSO predictions. S denotes spectral features, E denotes entropy features, and C denotes complexity features.

**Table 4:** Classification metrics for the feature configuration yielding the highest BA in each diagnostic comparison under the EO condition.

Comparison	Features	BA (%)	Sensitivity (%)	Specificity (%)	AUC	F1-Score (%)	Precision (%)
AD-HC	S	84.91 [76.09–92.37]	83.33 [71.43–92.86]	86.49 [75.68–97.30]	0.94 [0.88–0.98]	85.37 [76.31–92.68]	87.50 [78.26–97.14]
AD-MCI	S	66.90 [54.52–77.14]	73.81 [59.52–85.71]	60.00 [40.00–76.67]	0.73 [0.59–0.84]	72.94 [62.65–81.40]	72.09 [62.00–82.50]
MCI-HC	E	70.14 [58.38–79.91]	70.00 [53.33–86.67]	70.27 [54.05–83.78]	0.71 [0.58–0.83]	67.74 [55.17–78.12]	65.62 [53.12–78.57]

Note. The 95% CIs were estimated using 1000 stratified bootstrap resamples of the outer-loop LOSO predictions. S denotes spectral features, E denotes entropy features, and C denotes complexity features.

**Table 5:** Highest-performing feature configuration for each binary classification task under EC and EO conditions.

Comparison	Highest BA (EC)	BA (%)	Highest BA (EO)	BA (%)
AD-HC	S	79.99	S	84.91
AD-MCI	S + E	68.33	S	66.90
MCI-HC	S + E + C	63.15	E	70.14

Note. S denotes spectral features, E denotes entropy features, and C denotes complexity features.

**Table 6:** Number of retained features within individual feature domains following Stage-1 and Stage-2 feature selection for each diagnostic comparison under the EO and the EC conditions.

Comparison	Condition	Stage	Spectral	Entropy	Complexity
AD-HC (*n* = 79 folds)	EO	Stage 1	58.8 ± 10.2	104.6 ± 13.3	90.0 ± 9.2
		Stage 2	12.9 ± 2.9	20.5 ± 5.3	15.2 ± 3.1
	EC	Stage 1	62.4 ± 12.8	111.0 ± 14.7	91.2 ± 9.8
		Stage 2	10.6 ± 3.5	23.0 ± 6.3	19.1 ± 5.7
AD-MCI (*n* = 72 folds)	EO	Stage 1	77.3 ± 12.9	109.1 ± 14.1	85.2 ± 9.6
		Stage 2	25.1 ± 6.0	23.3 ± 6.5	25.7 ± 5.3
	EC	Stage 1	83.5 ± 14.0	100.2 ± 12.9	93.5 ± 10.0
		Stage 2	19.5 ± 4.9	17.9 ± 5.5	24.8 ± 4.7
MCI-HC (*n* = 67 folds)	EO	Stage 1	75.9 ± 11.8	100.0 ± 12.1	87.3 ± 10.5
		Stage 2	22.3 ± 4.6	20.6 ± 5.0	22.8 ± 5.0
	EC	Stage 1	84.6 ± 12.9	103.8 ± 12.8	90.8 ± 9.6
		Stage 2	19.7 ± 5.4	18.2 ± 5.2	21.5 ± 5.5

**Table 7:** Most frequently selected electrodes accounting for at least 50% of cumulative feature selections across LOSO folds under EC and EO conditions.

Comparison	Feature Set	EC	EO
AD-HC	Spectral	T3, T4, T6, Fz	T4, F8, P3
	Entropy	C4, Fp1, T6, P3, F7, Cz	F7, T6, P3, T4
	Complexity	T3, P3, F8	T5, F8, P3, T4
AD-MCI	Spectral	T4, Fz, P3, C4, C3	F7, Pz, Cz, T5, T4, P3
	Entropy	F4, Fz, F3, P4, F7	Cz, T5, F7, T3, Pz, O2
	Complexity	Cz, P3, T4, F8, C3, P4	Pz, F8, F7, C4, O2
MCI-HC	Spectral	P3, T3, C3, F8, O2	F3, T3, F8, T4, Fp2, F4
	Entropy	F3, T4, T3, Fp2, C4, Fz	F4, C4, F3, P4, T4
	Complexity	Fp1, C4, F7, F3, Cz, T3	C4, T4, P4, Fz, F7

Note. Electrodes are listed in descending order of relative selection proportions.

## Data Availability

The CAUEEG dataset used in this study was obtained from the official repository: https://github.com/ipis-mjkim/caueeg-dataset (accessed on 17 September 2026). Access to the dataset requires submission of an application form and approval from the local ethics committee. Following review of the application, the data providers provide access to the dataset via a download link and password.

## Supplementary Materials

The following supporting information can be downloaded at https://www.mdpi.com/article/10.3390/e28091034/s1, Table S1. Input parameters of the entropy and complexity feature-extraction algorithms used throughout this study. Table S2. Classification performance of individual and combined feature families under the eyes-closed condition. Values are presented as point estimates, with the corresponding 95% confidence intervals (CIs) shown in brackets. AD, Alzheimer’s disease; MCI, mild cognitive impairment; HC, healthy controls; EC, eyes-closed; BA, balanced accuracy; AUC, area under the receiver operating characteristic curve; S, spectral features; E, entropy features; C, complexity features. Table S3. Classification performance of individual and combined feature families under the eyes-open condition. Values are presented as point estimates, with the corresponding 95% confidence intervals (CIs) shown in brackets. AD, Alzheimer’s disease; MCI, mild cognitive impairment; HC, healthy controls; EO eyes-open; BA, balanced accuracy; AUC, area under the receiver operating characteristic curve; S, spectral features; E, entropy features; C, complexity features. Figure S1. Scalp maps showing the electrode-wise distribution of spectral metrics selections under EC conditions for the pairwise group comparisons AD-HC, AD-MCI, and MCI-HC. Percentages within each electrode indicate the share of total spectral metric selections attributed to that channel. The blue colour scale represents the relative selection frequency, with darker shades indicating larger contributions. Figure S2. Scalp maps showing the electrode-wise distribution of entropy metrics selections under EC conditions for the pairwise group comparisons AD-HC, AD-MCI, and MCI-HC. Percentages within each electrode indicate the share of total entropy metric selections attributed to that channel. The blue colour scale represents the relative selection frequency, with darker shades indicating larger contributions. Figure S3. Scalp maps showing the electrode-wise distribution of complexity metrics selections under EC conditions for the pairwise group comparisons AD-HC, AD-MCI, and MCI-HC. Percentages within each electrode indicate the share of total complexity metric selections attributed to that channel. The blue colour scale represents the relative selection frequency, with darker shades indicating larger contributions. Figure S4. Scalp maps showing the electrode-wise distribution of spectral metrics selections under EO conditions for the pairwise group comparisons AD-HC, AD-MCI, and MCI-HC. Percentages within each electrode indicate the share of total spectral metric selections attributed to that channel. The blue colour scale represents the relative selection frequency, with darker shades indicating larger contributions. Figure S5. Scalp maps showing the electrode-wise distribution of entropy metrics selections under EO conditions for the pairwise group comparisons AD-HC, AD-MCI, and MCI-HC. Percentages within each electrode indicate the share of total entropy metric selections attributed to that channel. The blue colour scale represents the relative selection frequency, with darker shades indicating larger contributions. Figure S6. Scalp maps showing the electrode-wise distribution of complexity metrics selections under EO conditions for the pairwise group comparisons AD-HC, AD-MCI, and MCI-HC. Percentages within each electrode indicate the share of total complexity metric selections attributed to that channel. The blue colour scale represents the relative selection frequency, with darker shades indicating larger contributions. References [65,66] are cited in Supplementary Table S1.

## Abbreviations

ACC	Accuracy	HJ-C	Hjorth complexity
AD	Alzheimer’s disease	HJ-M	Hjorth mobility
ADAS-Cog	Alzheimer’s Disease Assessment Scale-Cognitive Subscale	KFD	Katz fractal dimension
AE	Attention entropy	LZC	Lempel–Ziv complexity
AMIR	Auto mutual information rate	MCI	Mild cognitive impairment
BA	Balanced accuracy	MF	Median frequency
BubbleEn	Bubble entropy	MMSE	Mini-Mental State Examination
BW	Bandwidth	MRI	Magnetic resonance imaging
CAUEEG	Chung-Ang University Hospital EEG	MSE	Multiscale sample entropy
CDR	Clinical Dementia Rating	mvMSE	Multivariate multiscale sample entropy
CF	Centre frequency	PET	Positron emission tomography
CSF	Cerebrospinal fluid	PSD	Power spectral density
CTM	Central tendency measure	PW	Power
DFA	Detrended fluctuation analysis	RangeEn	Range entropy
EC	Eyes closed	AUC	Area under the receiver operating characteristic curve
EEG	Electroencephalography	RP	Relative power
EO	Eyes open	TNR	True negative rate
EoE	Entropy of entropy	TPESampler	Tree-structured Parzen estimator
FuzzyEn	Fuzzy entropy	TPR	True positive rate
HCs	Healthy controls	WE	Wavelet entropy
HFD	Higuchi fractal dimension	XFuzzyEn	Cross-fuzzy entropy
