# Supplementary material for: Impact of Recording Conditions and Feature Fusion on Dementia Classification Using Spectral, Entropy, and Complexity EEG Features
Source: Entropy (Basel). 2026 Sep 20;28(9):1034. doi: 10.3390/e28091034 (PMC13605554; doi:10.3390/e28091034)
Supplement: Supplementary file 1 [file entropy-28-01034-s001.zip › entropy-4493027-supplementary.pdf]

Supplementary Materials

This supplementary section provides detailed methodological information and complementary classification results for the spectral, entropy, and complexity feature domains under EC and EO conditions. Table S1 summarises the parameterisation of the entropy and complexity metrics, together with the methodological modifications introduced in this study and the rationale for these choices. Tables S2 and S3 report the binary classification performance for the AD–HC, AD–MCI, and MCI–HC comparisons under EC and EO conditions, respectively. Classification results are presented for each feature family (spectral, entropy, and complexity), for all pairwise domain combinations, and for the combined use of all three feature families. Figures S1–S3 show the scalp distribution of electrode-selection frequencies for the spectral, entropy, and complexity domains, respectively, under the EC condition, whereas Figures S4–S6 present the corresponding distributions under the EO condition in the same order.

Table S1. Input parameters of the entropy and complexity feature-extraction algorithms used throughout this study.

Feature	Literature basis / modification	Rationale
AMIR	Based on [21]. The previous study used 4096-sample signals with 64 bins; for the present 1000-sample signals, **16 *bins*** were used. The maximum delay was limited to **100 *samples*** with a delay step of 1	The number of bins was reduced in accordance with the shorter signal length, while the maximum delay was selected to represent a maximum temporal lag of approximately **0.5 s**.
CTM	Parameters were chosen based on [65]: ***threshold* = 0.5 × *SD (epoch)***.	No modification was applied.
BubbleEn	Parameters were chosen based on [32]: ***m* = *4, τ = 1, Logx* = *e***.	BubbleEn has been reported to be relatively insensitive to the embedding dimension, making it a nearly parameter-free estimator; therefore, the published setting was retained.
XFuzzyEn	Parameters were chosen based on [33]: ***m* = *2, τ = 1, r = 0.2, n*=*2, Logx* = *e***.	No modification was applied.
EoE	Based on [34], with ***τ = 10*** *and* ***S = 10***.	The number of amplitude slices was set to ***S* = *10*** following the previously reported configuration.
FuzzyEn	Based on [17]. The previously reported ***m* = 2** and ***r* = *0.25* × *SD*** were retained, whereas the fuzzy power parameter was changed from ***n* = *1*** **to** ***n*** **= 2**.	Increasing *n* gives greater emphasis to highly similar patterns and produces a sharper distinction between patterns with different degrees of similarity.
MSE	Based on [59]. The previously reported **r = 0.25** was retained, whereas **m** was increased from ***1 to 2***; scales ***1–10*** were analysed.	Although $m = 1$ has shown good statistical reproducibility for sufficiently long sequences, $m = 2$ was used here to retain sensitivity to higher-order temporal dynamics.
mvMSE	Parameters were chosen based on [66]: ***m* = *1*** **for all channels, *r* = *0.15*, *scales* = *1–10***, with the multivariate coarse-graining procedure.	No modification was applied.
RangeEn	Parameters were chosen based on [36]: ***m* = *2, τ = 1, r = 0.20***.	No modification was applied.

Abbreviations: *m*, embedding dimension; *τ*, time delay; *r*, tolerance threshold (expressed as a fraction of the signal's standard deviation unless otherwise stated); *S*, number of amplitude slices; *Logx*, logarithmic base; *SD*, standard deviation.

Table S2. Classification performance of individual and combined feature families under the eyes-closed condition. Values are presented as point estimates, with the corresponding 95% confidence intervals (CIs) shown in brackets. AD, Alzheimer's disease; MCI, mild cognitive impairment; HC, healthy controls; EC, eyes-closed; BA, balanced accuracy; AUC, area under the receiver operating characteristic curve; S, spectral features; E, entropy features; C, complexity features.

Comparison	Feature	BA (%)	Sensitivity (%)	Specificity (%)	AUC	F1-score (%)	Precision (%)
**AD-HC**	*S*	79.99 [71.01-88.96]	76.19 [61.90-88.10]	83.78 [70.27-94.59]	0.87 [0.78-0.95]	80.00 [70.13-89.16]	84.21 [74.42-94.29]
**AD-HC**	*E*	71.72 [62.26-81.72]	78.57 [66.67-90.48]	64.86 [48.65-81.08]	0.75 [0.64-0.86]	75.00 [66.65-83.72]	71.74 [62.96-81.82]
**AD-HC**	*C*	78.64 [69.50-86.59]	76.19 [61.90-88.10]	81.08 [70.20-91.89]	0.84 [0.75-0.92]	79.01 [69.23-87.18]	82.05 [72.50-91.90]
**AD-HC**	*S + E*	69.82 [59.49-79.13]	66.67 [52.38-78.57]	72.97 [59.46-86.49]	0.79 [0.68-0.89]	70.00 [58.97-79.52]	73.68 [62.50-85.29]
**AD-HC**	*S + C*	78.64 [69.50-87.61]	76.19 [61.90-88.10]	81.08 [67.57-91.89]	0.83 [0.73-0.91]	79.01 [69.23-87.80]	82.05 [71.43-91.89]
**AD-HC**	*E + C*	73.39 [64.09-82.53]	73.81 [59.52-85.71]	72.97 [59.46-86.49]	0.81 [0.70-0.90]	74.70 [65.00-83.52]	75.61 [65.91-86.84]
**AD-HC**	*S + E + C*	73.55 [63.06-82.69]	71.43 [57.14-83.33]	75.68 [59.46-89.19]	0.82 [0.73-0.91]	74.07 [63.76-83.33]	76.92 [65.85-87.88]
**AD-MCI**	*S*	67.62 [56.18-78.33]	78.57 [64.29-90.48]	56.67 [40.00-73.33]	0.75 [0.63-0.87]	75.00 [65.06-83.52]	71.74 [62.96-81.40]
**AD-MCI**	*E*	64.05 [52.85-75.24]	71.43 [57.14-83.39]	56.67 [40.00-73.33]	0.72 [0.59-0.84]	70.59 [59.52-80.00]	69.77 [60.47-80.49]
**AD-MCI**	*C*	64.29 [53.09-75.01]	78.57 [64.29-90.48]	50.00 [30.00-66.67]	0.65 [0.50-0.78]	73.33 [63.63-81.72]	68.75 [60.42-78.05]
**AD-MCI**	*S + E*	68.33 [57.61-78.58]	83.33 [71.43-92.86]	53.33 [36.67-70.00]	0.66 [0.51-0.79]	76.92 [68.68-84.44]	71.43 [63.64-80.01]
**AD-MCI**	*S + C*	67.62 [56.90-78.33]	78.57 [64.29-90.48]	56.67 [40.00-73.33]	0.76 [0.64-0.87]	75.00 [65.88-83.15]	71.74 [63.46-81.08]
**AD-MCI**	*E + C*	65.95 [55.23-77.38]	78.57 [64.29-88.15]	53.33 [36.67-73.33]	0.61 [0.45-0.75]	74.16 [64.52-82.36]	70.21 [62.00-80.00]
**AD-MCI**	*S + E + C*	65.24 [53.57-76.68]	73.81 [59.52-85.71]	56.67 [39.92-76.67]	0.69 [0.55-0.83]	72.09 [61.73-81.40]	70.45 [61.11-82.05]
**MCI-HC**	*S*	58.47 [47.43-69.92]	46.67 [30.00-63.33]	70.27 [56.76-83.78]	0.62 [0.49-0.77]	50.91 [34.62-65.52]	56.00 [40.91-72.74]
**MCI-HC**	*E*	57.43 [45.05-68.56]	50.00 [33.33-66.67]	64.86 [48.65-81.08]	0.58 [0.45-0.71]	51.72 [35.71-64.73]	53.57 [38.88-68.18]
**MCI-HC**	*C*	47.34 [34.95-59.82]	43.33 [23.33-63.33]	51.35 [35.14-67.57]	0.49 [0.36-0.64]	42.62 [27.11-56.25]	41.94 [27.99-55.18]
**MCI-HC**	*S + E*	55.77 [44.41-67.84]	46.67 [30.00-63.33]	64.86 [48.65-78.38]	0.58 [0.44-0.71]	49.12 [33.33-63.33]	51.85 [37.03-66.70]
**MCI-HC**	*S + C*	57.75 [45.67-69.82]	53.33 [36.67-70.00]	62.16 [45.95-78.38]	0.60 [0.46-0.74]	53.33 [38.46-66.67]	53.33 [40.00-67.86]

Comparison	Feature	BA (%)	Sensitivity (%)	Specificity (%)	AUC	F1-score (%)	Precision (%)
**MCI-HC**	*E + C*	55.45 [44.31-66.49]	43.33 [26.67-63.33]	67.57 [54.05-81.08]	0.56 [0.43-0.69]	47.27 [30.77-62.07]	52.00 [36.66-68.00]
**MCI-HC**	*S + E + C*	63.15 [51.48-74.19]	53.33 [36.67-70.00]	72.97 [56.76-86.49]	0.64 [0.51-0.76]	57.14 [42.11-70.77]	61.54 [46.67-76.92]

Table S3. Classification performance of individual and combined feature families under the eyes-open condition. Values are presented as point estimates, with the corresponding 95% confidence intervals (CIs) shown in brackets. AD, Alzheimer's disease; MCI, mild cognitive impairment; HC, healthy controls; EO eyes-open; BA, balanced accuracy; AUC, area under the receiver operating characteristic curve; S, spectral features; E, entropy features; C, complexity features.

Comparison	Features	BA (%)	Sensitivity (%)	Specificity (%)	AUC	F1-score (%)	Precision (%)
**AD-HC**	*S*	84.91 [76.09-92.37]	83.33 [71.43-92.86]	86.49 [75.68-97.30]	0.94 [0.88-0.98]	85.37 [76.31-92.68]	87.50 [78.26-97.14]
**AD-HC**	*E*	74.74 [64.58-83.72]	73.81 [61.90-85.71]	75.68 [62.16-89.19]	0.79 [0.68-0.88]	75.61 [65.75-84.71]	77.50 [67.44-88.24]
**AD-HC**	*C*	73.07 [64.41-82.21]	78.57 [66.67-90.48]	67.57 [51.35-81.08]	0.76 [0.65-0.87]	75.86 [67.44-84.09]	73.33 [65.21-83.34]
**AD-HC**	*S + E*	77.99 [68.85-86.97]	85.71 [73.81-95.24]	70.27 [54.05-83.78]	0.86 [0.77-0.94]	80.90 [72.94-88.37]	76.60 [68.00-86.05]
**AD-HC**	*S + C*	80.69 [71.06-88.66]	85.71 [73.81-95.24]	75.68 [59.46-89.19]	0.91 [0.83-0.97]	82.76 [74.42-90.12]	80.00 [70.59-89.74]
**AD-HC**	*E + C*	78.64 [69.65-86.42]	76.19 [61.90-88.10]	81.08 [67.57-91.89]	0.79 [0.68-0.89]	79.01 [69.14-86.96]	82.05 [72.50-91.89]
**AD-HC**	*S + E + C*	71.40 [61.87-81.02]	83.33 [71.43-92.86]	59.46 [43.24-75.68]	0.83 [0.73-0.91]	76.09 [68.23-84.00]	70.00 [62.06-79.17]
**AD-MCI**	*S*	66.90 [54.52-77.14]	73.81 [59.52-85.71]	60.00 [40.00-76.67]	0.73 [0.59-0.84]	72.94 [62.65-81.40]	72.09 [62.00-82.50]
**AD-MCI**	*E*	66.19 [54.04-77.14]	69.05 [54.76-81.01]	63.33 [46.67-80.00]	0.67 [0.52-0.80]	70.73 [58.66-80.44]	72.50 [61.90-84.21]
**AD-MCI**	*C*	57.14 [45.95-68.33]	64.29 [50.00-76.19]	50.00 [33.33-70.00]	0.55 [0.41-0.69]	64.29 [53.33-73.57]	64.29 [54.84-75.00]
**AD-MCI**	*S + E*	57.62 [45.71-69.53]	61.90 [47.62-76.19]	53.33 [33.33-73.33]	0.61 [0.46-0.74]	63.41 [51.35-73.57]	65.00 [54.35-75.76]
**AD-MCI**	*S + C*	66.43 [55.23-77.14]	76.19 [61.90-88.10]	56.67 [40.00-73.33]	0.68 [0.54-0.81]	73.56 [63.53-81.82]	71.11 [62.49-80.56]
**AD-MCI**	*E + C*	62.38 [51.19-73.57]	71.43 [57.14-83.33]	53.33 [33.33-70.00]	0.59 [0.45-0.74]	69.77 [59.26-78.66]	68.18 [59.18-78.57]
**AD-MCI**	*S + E + C*	60.00 [48.57-71.90]	66.67 [52.38-80.95]	53.33 [36.67-73.33]	0.57 [0.43-0.71]	66.67 [55.41-76.20]	66.67 [57.14-76.93]
**MCI-HC**	*S*	65.14 [53.78-76.49]	60.00 [40.00-76.67]	70.27 [56.69-83.78]	0.68 [0.56-0.80]	61.02 [46.42-73.69]	62.07 [49.96-76.67]

Comparison	Features	BA (%)	Sensitivity (%)	Specificity (%)	AUC	F1-score (%)	Precision (%)
**MCI-HC**	*E*	70.14 [58.38-79.91]	70.00 [53.33-86.67]	70.27 [54.05-83.78]	0.71 [0.58-0.83]	67.74 [55.17-78.12]	65.62 [53.12-78.57]
**MCI-HC**	*C*	52.66 [40.89-64.64]	56.67 [40.00-73.33]	48.65 [32.43-64.86]	0.58 [0.43-0.71]	51.52 [38.60-63.16]	47.22 [36.36-58.62]
**MCI-HC**	*S + E*	52.75 [39.95-64.82]	43.33 [26.67-60.00]	62.16 [45.95-75.68]	0.55 [0.41-0.68]	45.61 [30.18-59.66]	48.15 [33.33-63.34]
**MCI-HC**	*S + C*	69.82 [59.09-80.95]	66.67 [50.00-83.33]	72.97 [59.46-86.49]	0.72 [0.60-0.84]	66.67 [53.73-79.37]	66.67 [54.29-80.65]
**MCI-HC**	*E + C*	59.41 [47.11-70.45]	56.67 [39.92-73.33]	62.16 [45.95-75.74]	0.74 [0.61-0.84]	55.74 [41.36-68.50]	54.84 [42.10-67.86]
**MCI-HC**	*S + E + C*	66.17 [55.14-77.21]	56.67 [40.00-73.33]	75.68 [62.09-89.19]	0.71 [0.58-0.82]	60.71 [46.15-74.58]	65.38 [51.61-80.00]

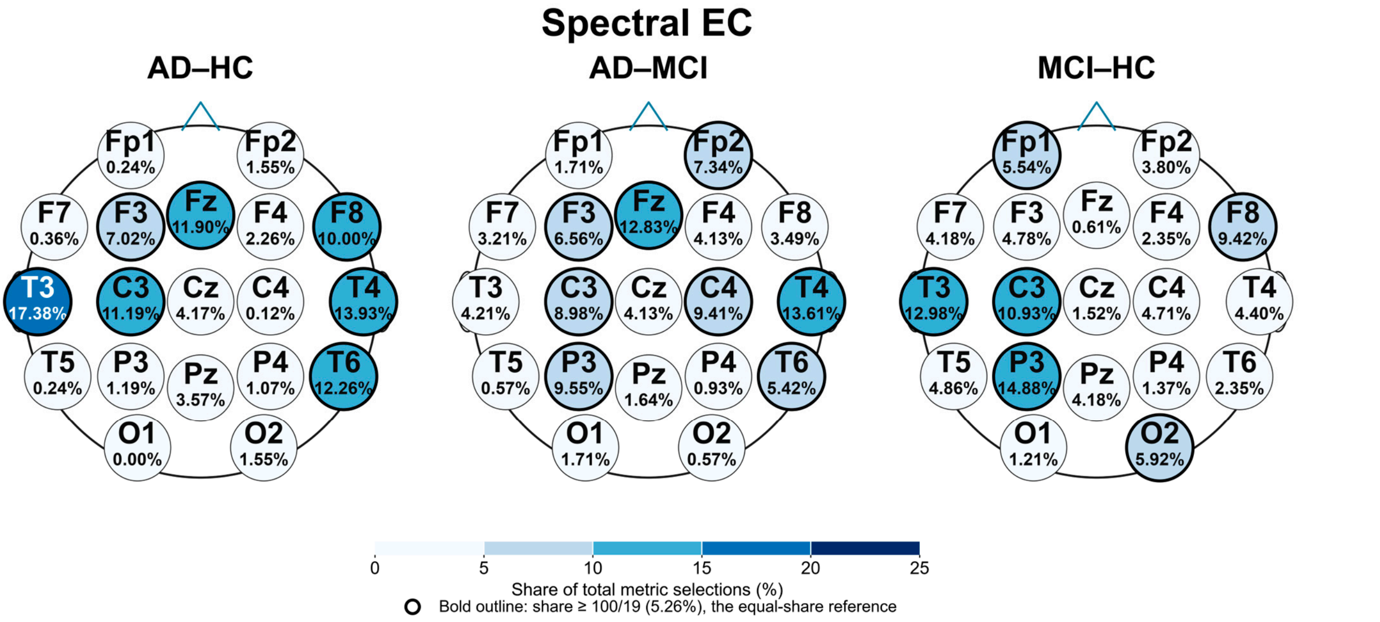

Figure S1. Scalp maps showing the electrode-wise distribution of spectral metrics selections under EC conditions for the pairwise group comparisons AD–HC, AD–MCI, and MCI–HC. Percentages within each electrode indicate the share of total spectral metric selections attributed to that channel. The blue colour scale represents the relative selection frequency, with darker shades indicating larger contributions.

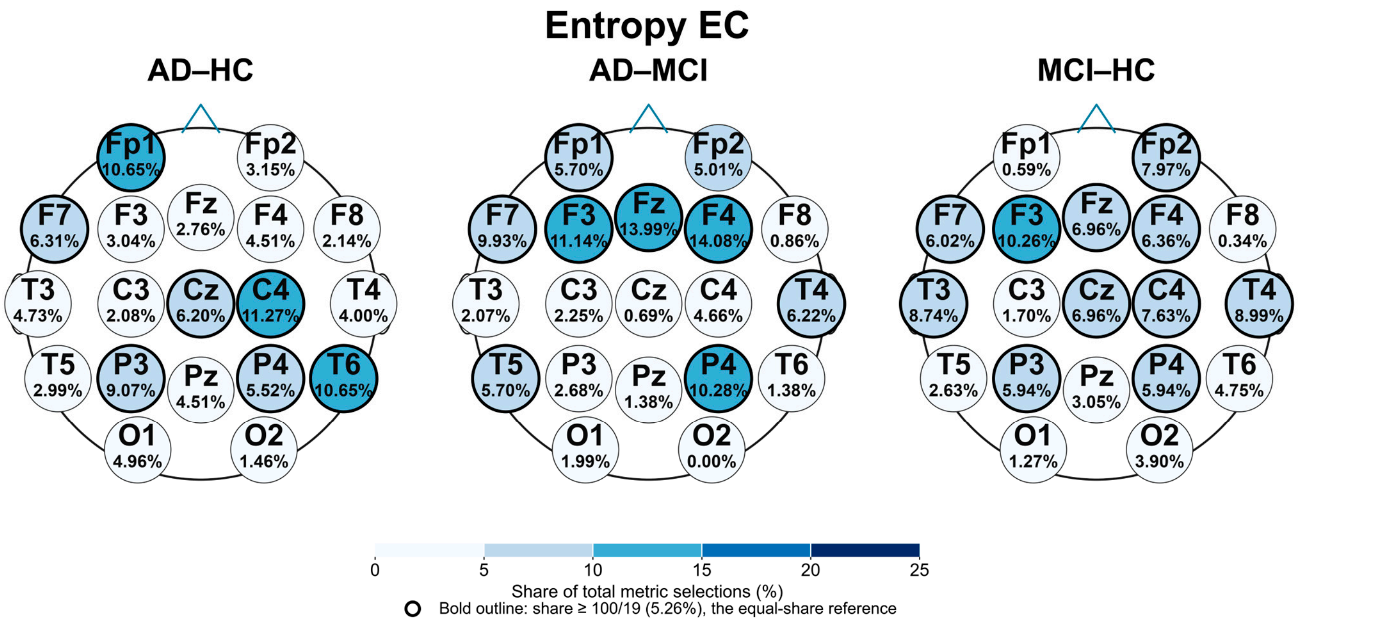

Figure S2. Scalp maps showing the electrode-wise distribution of entropy metrics selections under EC conditions for the pairwise group comparisons AD–HC, AD–MCI, and MCI–HC. Percentages within each electrode indicate the share of total entropy metric selections attributed to that channel. The blue colour scale represents the relative selection frequency, with darker shades indicating larger contributions.

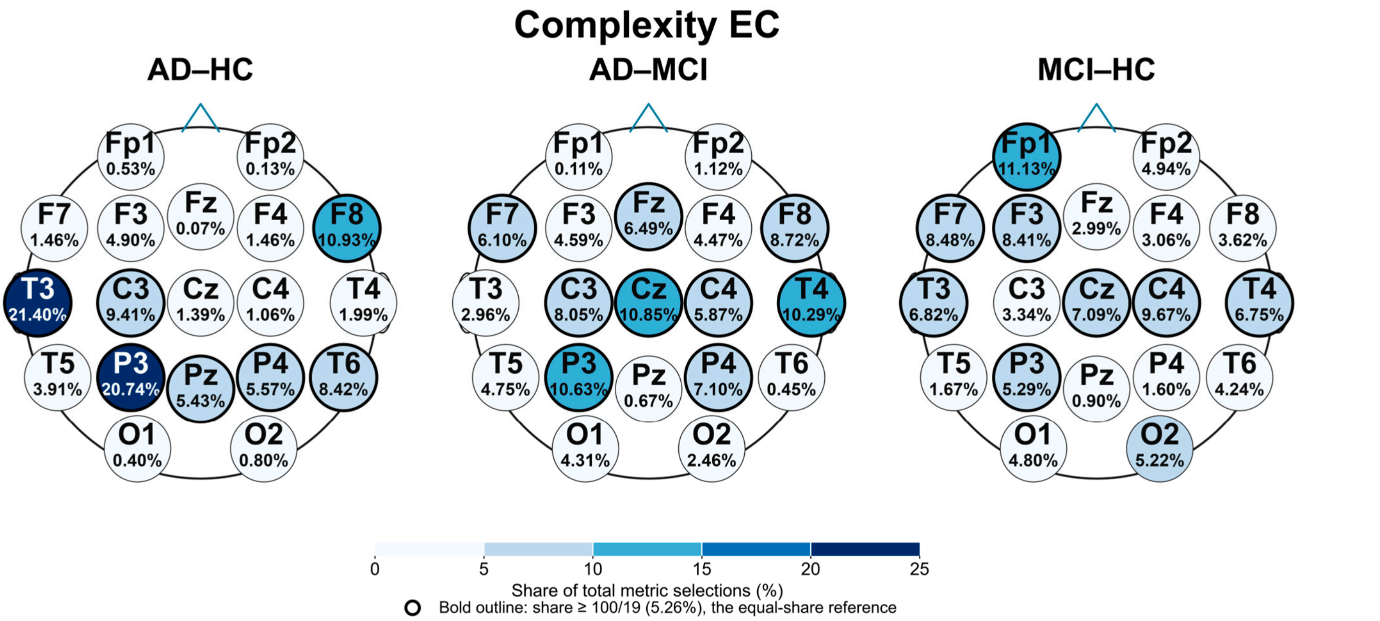

Figure S3. Scalp maps showing the electrode-wise distribution of complexity metrics selections under EC conditions for the pairwise group comparisons AD–HC, AD–MCI, and MCI–HC. Percentages within each electrode indicate the share of total complexity metric selections attributed to that channel. The blue colour scale represents the relative selection frequency, with darker shades indicating larger contributions.

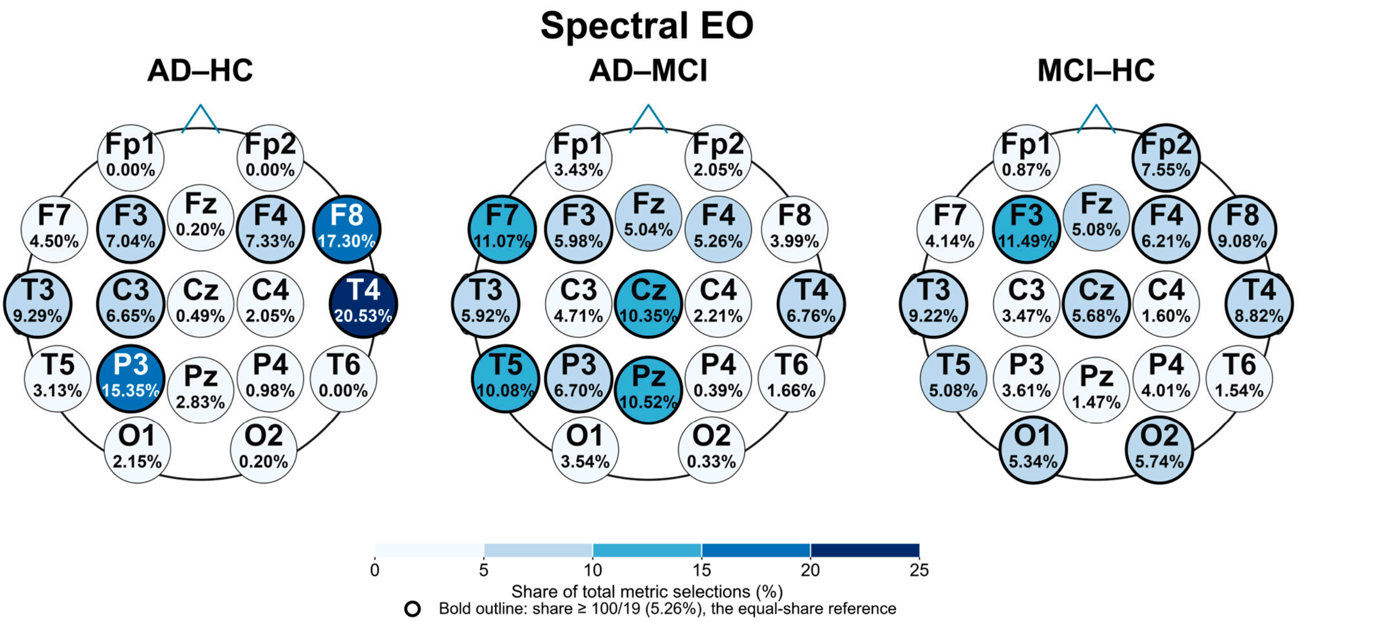

Figure S4. Scalp maps showing the electrode-wise distribution of spectral metrics selections under EO conditions for the pairwise group comparisons AD–HC, AD–MCI, and MCI–HC. Percentages within each electrode indicate the share of total spectral metric selections attributed to that channel. The blue colour scale represents the relative selection frequency, with darker shades indicating larger contributions.

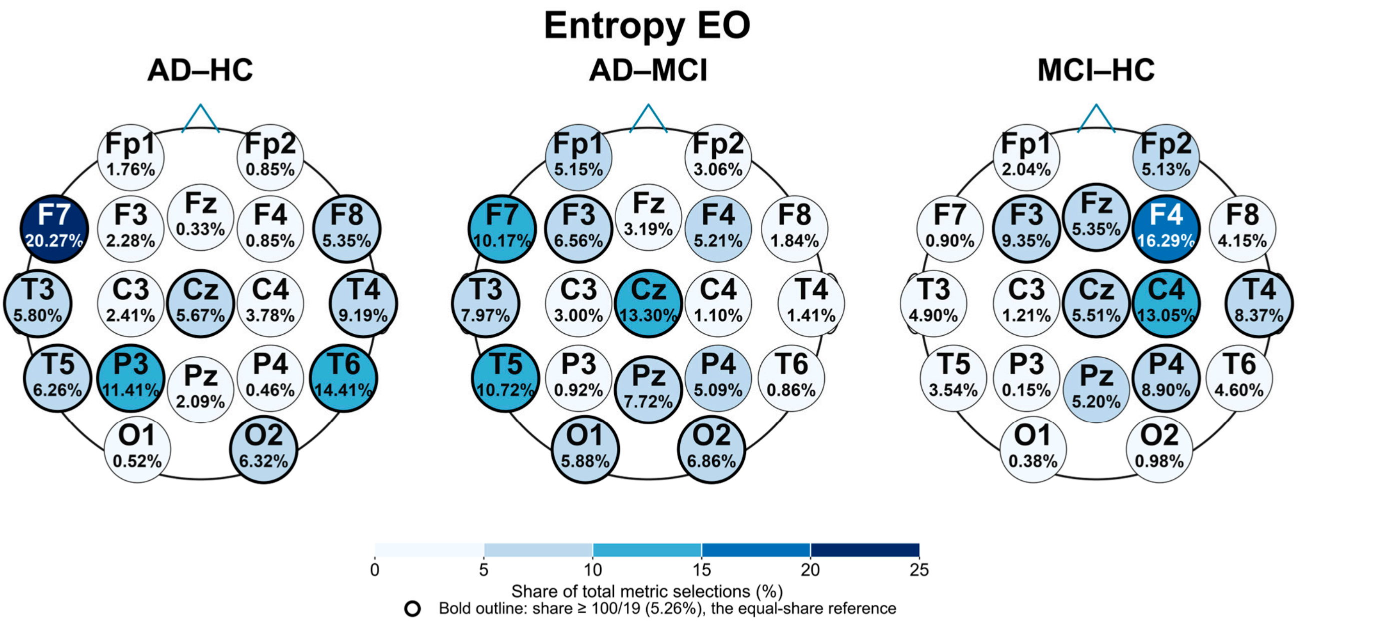

Figure S5. Scalp maps showing the electrode-wise distribution of entropy metrics selections under EO conditions for the pairwise group comparisons AD–HC, AD–MCI, and MCI–HC. Percentages within each electrode indicate the share of total entropy metric selections attributed to that channel. The blue colour scale represents the relative selection frequency, with darker shades indicating larger contributions.

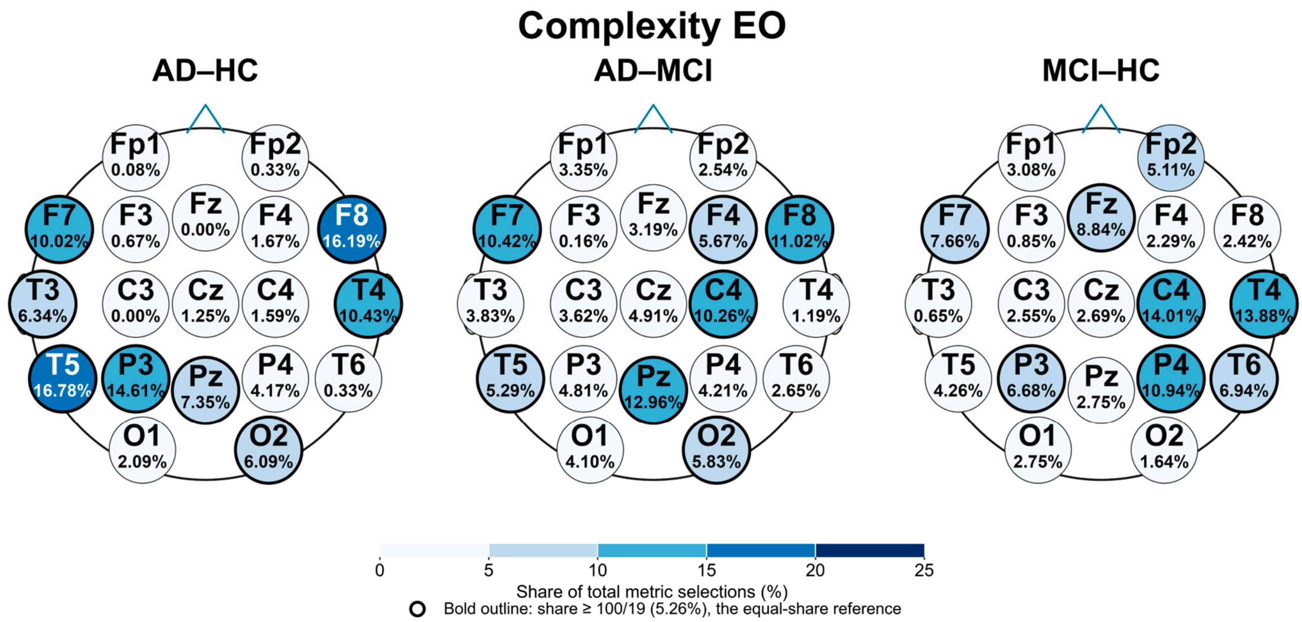

Figure S6. Scalp maps showing the electrode-wise distribution of complexity metrics selections under EO conditions for the pairwise group comparisons AD–HC, AD–MCI, and MCI–HC. Percentages within each electrode indicate the share of total complexity metric selections attributed to that channel. The blue colour scale represents the relative selection frequency, with darker shades indicating larger contributions.
